# *Yersinia pseudotuberculosis* doxycycline tolerance strategies include modulating expression of genes involved in cell permeability and tRNA modifications

**DOI:** 10.1371/journal.ppat.1010556

**Published:** 2022-05-16

**Authors:** Hector S. Alvarez-Manzo, Robert K. Davidson, Jasper Van Cauwelaert de Wyels, Katherine L. Cotten, Benjamin H. Nguyen, Melody Xiao, Zeyu Zhu, Jon Anthony, Tim van Opijnen, Kimberly Michele Davis

**Affiliations:** 1 W. Harry Feinstone Department of Molecular Microbiology and Immunology Johns Hopkins Bloomberg School of Public Health, Baltimore, Maryland, United States of America; 2 Department of Biology, Boston College, Chestnut Hill, Massachusetts, United States of America; Stanford University School of Medicine, UNITED STATES

## Abstract

Antibiotic tolerance is typically associated with a phenotypic change within a bacterial population, resulting in a transient decrease in antibiotic susceptibility that can contribute to treatment failure and recurrent infections. Although tolerant cells may emerge prior to treatment, the stress of prolonged antibiotic exposure can also promote tolerance. Here, we sought to determine how *Yersinia pseudotuberculosis* responds to doxycycline exposure, to then verify if these gene expression changes could promote doxycycline tolerance in culture and in our mouse model of infection. Only four genes were differentially regulated in response to a physiologically-relevant dose of doxycycline: *osmB* and *ompF* were upregulated, *tusB* and *cnfy* were downregulated; differential expression also occurred during doxycycline treatment in the mouse. *ompF*, *tusB* and *cnfy* were also differentially regulated in response to chloramphenicol, indicating these could be general responses to ribosomal inhibition. *cnfy* has previously been associated with persistence and was not a major focus here. We found deletion of the OmpF porin resulted in increased antibiotic accumulation, suggesting expression may promote diffusion of doxycycline out of the cell, while OsmB lipoprotein had a minor impact on antibiotic permeability. Overexpression of *tusB* significantly impaired bacterial survival in culture and in the mouse, suggesting that tRNA modification by *tusB*, and the resulting impacts on translational machinery, promotes survival during treatment with an antibiotic classically viewed as bacteriostatic. We believe this may be the first observation of bactericidal activity of doxycycline under physiological conditions, which was revealed by reversing *tusB* downregulation.

## Author summary

The initial goal of this study was to determine how *Yersinia pseudotuberculosis* changes transcriptionally in response to doxycycline exposure, to then determine if these transcriptional changes are required to survive prolonged drug exposure. Our data suggests that porin expression is not just involved in antibiotic entry into bacterial cells, and may also promote passive diffusion of doxycycline out of the cell. Unexpectedly, we also found that overexpression of *tusB*, a gene product involved in tRNA modifications, resulted in potent doxycycline bactericidal activity at the typical minimum inhibitory concentration for *Y*. *pseudotuberculosis*. Doxycycline is classically viewed as a bacteriostatic antibiotic, which reversibly binds ribosomes, resulting in bacterial growth inhibition. However, growing evidence suggests that bacteriostatic antibiotics can also have bactericidal activity. We believe our results may be the first demonstration of the bactericidal activity of doxycycline under physiological conditions; with a bacterial pathogen, at a drug concentration that can be obtained within mouse and human tissues.

## Introduction

Antibiotics have saved countless lives, however since the introduction of penicillin as a clinical treatment in the 1940s, researchers and clinicians observed that bacterial pathogens may have inherent resistance or decreased susceptibility to antibiotics [1,2]. This can occur through many different molecular mechanisms, which are broadly grouped into antibiotic resistance and antibiotic tolerance mechanisms. Antibiotic resistance is typically associated with a genetic change within a bacterial population, which allows cells to not only survive, but continue to replicate, in the presence of inhibitory concentrations of antibiotics [3,4]. In contrast, antibiotic tolerance is typically associated with transient, phenotypic changes, though genetic changes can also contribute to tolerance [3–6]. During antibiotic treatment, tolerant cells survive in the presence of inhibitory concentrations of drug, but do not divide until drug concentrations decrease to subinhibitory levels [3,4,7]. Antibiotic tolerance can be caused by environmental stressors imparted on the bacterial population prior to antibiotic exposure, but tolerance can also emerge in response to exposure to the drug itself [8–11]. Generally, antibiotic tolerance is associated with transient slowed growth [8,12,13], which renders antibiotic action less effective. Although resistance is more heavily studied, tolerance also plays a major role in antibiotic treatment failure, and it is critical to better understand tolerance processes to develop novel therapeutic options that more effectively eliminate bacterial infection.

Antibiotics are generally divided into two categories: bactericidal and bacteriostatic drugs; where bactericidal drugs promote killing of bacterial cells, and bacteriostatic drugs promote bacterial growth inhibition. Bacteriostatic drugs then rely on the host immune system to clear growth-arrested cells to resolve infection. Growing evidence suggests these definitions do not always apply, that the drug concentrations obtained dictate the activity of the drug, and that classically defined bacteriostatic drugs can also exhibit bactericidal activity [14–16]. While definitions of antibiotic resistance and tolerance readily apply to bactericidal activity, drugs acting in a bacteriostatic manner inherently arrest growth of bacterial populations, and so all bacterial populations would be considered ‘tolerant’ to bacteriostatic activity. This definition of tolerance then only applies to bacteriostatic drugs that have bactericidal activity under a particular condition [14,16,17]. This is an important distinction for this study, which focuses on doxycycline treatment. Doxycycline was chosen for these studies because it is used to treat *Yersinia* infections, we have an established mouse model to study its efficacy, and fluorescent reporters for tracking doxycycline exposure [18–23]. Doxycycline is a classical bacteriostatic antibiotic of the tetracycline class that binds reversibly to 16S rRNA [15,24,25]. Two different mechanisms are known to promote doxycycline resistance: expression of efflux pumps that lower intracellular drug concentration, and production of proteins that protect ribosomes from doxycycline binding [15,24]. Doxycycline tolerance has not been explored previously, because doxycycline isn’t thought to have bactericidal activity. However, in this study, we have observed bactericidal activity of doxycycline during treatment of a mutant strain. We believe this may be the first description of bactericidal activity of doxycycline under physiological drug concentrations, which may be related to reduced ribosomal activity during *tusB* overexpression.

Bacterial infections are particularly difficult to treat when bacteria spread to the bloodstream and colonize deep tissue sites, such as the spleen. It can be difficult to deliver inhibitory concentrations of antibiotics to deep tissues, and it can be challenging to eliminate the entire bacterial population [18,26,27]. Subsets of bacteria may survive due to exposure to subinhibitory concentrations of antibiotics, and subsets of bacteria may express sets of genes that render them less susceptible to antibiotics. When a subpopulation of bacteria emerges with increased antibiotic tolerance, this is termed antibiotic persistence, and the subpopulation is comprised of persister cells [4,7]. *Yersinia pseudotuberculosis* is known to cause long-term infections, associated with both intestinal and deep tissue sites, but it remained unclear if antibiotic persistence is underlying these clinical observations [28–30]. Our recent study showed that approximately 10% of the *Y*. *pseudotuberculosis* population replicating in the spleen will survive a single inhibitory dose of doxycycline (Dox) [18]. Dox was chosen for these experiments because it should be an effective treatment for *Yersinia* infection [19,23,31]. However, we found this bacterial subpopulation persisted in host tissues ~48 hours until the Dox level decreased, and then resumed growth, which defines these cells as an antibiotic-tolerant subpopulation of persister cells [3,32]. Stressed bacterial cells expressing the nitric oxide detoxifying gene, *hmp*, were enriched among the surviving cells, indicating NO stress may cause a change in bacteria that promotes survival [18]. However, only ~40% of surviving cells were Hmp^+^, suggesting additional pathways also contribute to survival. Exposure to subinhibitory doses of antibiotics can also promote survival by altering gene expression in the surviving cells [8,9]. Since this surviving bacterial population is exposed to inhibitory concentrations of Dox for ~48 hours prior to resuming growth, we hypothesized that phenotypic changes in response to Dox may also contribute to survival.

In this study we sought to determine if differential expression of specific genes alters the doxycycline susceptibility of *Y*. *pseudotuberculosis*. We performed RNA-seq to identify genes with altered expression during exposure to Dox, and constructed deletion and overexpression strains to determine the role of these gene products in *Y*. *pseudotuberculosis* doxycycline susceptibility. We show here that loss of expression of two outer membrane proteins, OmpF and OsmB, altered doxycycline accumulation, although this had minimal impact on bacterial survival. We also show that heightened expression of *tusB*, whose gene product is predicted to increase translational fidelity by stabilizing the wobble position of tRNAs [33,34], significantly increases Dox sensitivity.

## Results

### *ompF*, *osmB*, *CNFy*, and *tusB* are differentially regulated in response to doxycycline

Our recent study showed there is a subpopulation of *Y*. *pseudotuberculosis* that survives doxycycline (Dox) treatment in the mouse spleen, and host-derived nitric oxide initially promotes survival of stressed cells [18]. This subpopulation persists within host tissues for 48 hours before Dox concentrations wane and the bacterial population resumes growth. We hypothesized there may be phenotypic changes that occur in response to Dox, which promote bacterial survival in the hours after treatment.

**Fig 1:**
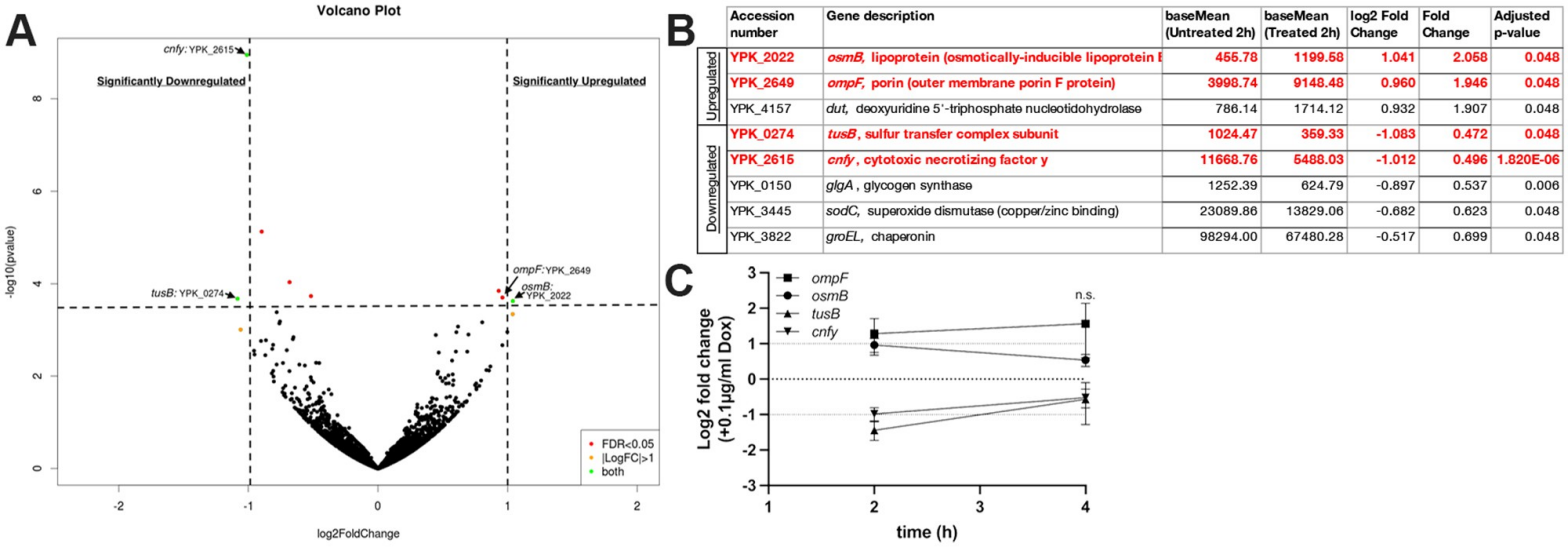
*ompF*, *osmB*, *CNFy*, and *tusB* are differentially regulated in response to doxycycline. Cultures of WT *Y*. *pseudotuberculosis* were incubated in the presence or absence of 0.1μg/ml Dox and bacterial transcript levels were compared by RNA-seq. Significant changes in transcript levels were detected at 2h post-treatment by DESeq2 analysis. **(A)** Volcano plot of aligned genes, each dot represents one gene. Horizontal dotted line: adjusted p value of 0.05, genes above this line are considered significantly altered. X-axis: log2 fold change in transcript levels. Vertical lines mark 2-fold changes, genes outside these lines are differentially regulated more than 2-fold. Hits of interest are highlighted. **(B)** Table of significant hits, 2h post-treatment. Hits of interest (differentially regulated ~2-fold) are highlighted in red. Base mean values represent 4 biological replicates. Log2 fold change and fold change are shown with adjusted p-value (adjusted based on gene length). **(C)** qRT-PCR validation of RNA-seq results. Cultures were prepared as described above, and bacterial transcripts were detected by qRT-PCR. Log2 fold change values were calculated relative to untreated cells, horizontal dotted lines depict average values for untreated cells (0log2), and 2-fold changes. Data represents four biological replicates. Statistics: **(C)** Two-way ANOVA with Tukey’s multiple comparison test, comparisons made relative to untreated cells. n.s.: not significant.

Using a fluorescent transcriptional reporter, we have approximated the concentrations of Dox in the spleen at 0.1μg/ml-1μg/ml during this period of growth inhibition following a single injection of 40mg/kg Dox [18]. To determine if there are specific pathways upregulated or downregulated during exposure to Dox, we initially chose to expose bacteria to 0.1μg/ml Dox. All bacterial cells would be exposed to at least this concentration within mouse tissues, and this concentration was sufficient for maximal de-repression of the *tet* operon [18], so we expected to see many transcriptional changes. We initially tested several different genes in known stress response pathways (ROS, RNS, general stress) to determine if these were differentially regulated in Dox-exposed *Y*. *pseudotuberculosis* in culture, but did not find significant changes (S1 Fig). We then took a global approach and utilized RNA-Seq to determine if there are specific pathways that would be up- or downregulated during exposure to 0.1μg/ml Dox. We exposed broth-grown bacteria to Dox and isolated bacterial RNA after 2h (hours, h) and 4h Dox exposure; treated and untreated cultures were grown in parallel. Samples were prepared for sequencing using a RNAtag-seq approach [35] and aligned to the *Y*. *pseudotuberculosis* genome. Although this concentration of Dox strongly modulates the TetR *tet* repressor [18], very few transcriptional changes occurred during antibiotic exposure *in vitro*, likely due to the low Dox concentration (Fig 1A). We identified 8 genes that were differentially regulated after 2h Dox treatment (Fig 1B), and there were no significant changes after 4h treatment, suggesting that transcriptionally, cells had returned to baseline. Of the 8 genes, only two genes were upregulated ~2 fold *(osmB*, *ompF*), and two genes were downregulated ~2 fold *(tusB*, *cnfy*), and so these 4 genes were selected for downstream analyses (highlighted in red, Fig 1B). We confirmed these 4 genes were differentially regulated after 2h Dox exposure by prepping additional bacterial samples for qRT-PCR, and also showed that gene expression changes were no longer observed after 4h Dox exposure (Fig 1C). These results were surprising; based on previous studies we had expected to see increased expression of efflux pumps and decreased expression of porins [36–38], instead we saw upregulation of a porin (*ompF*) and no change in efflux pump expression.

### *ompF*, *osmB*, *CNFy*, and *tusB* are differentially regulated in response to doxycycline *in vivo*

**Fig 2:**
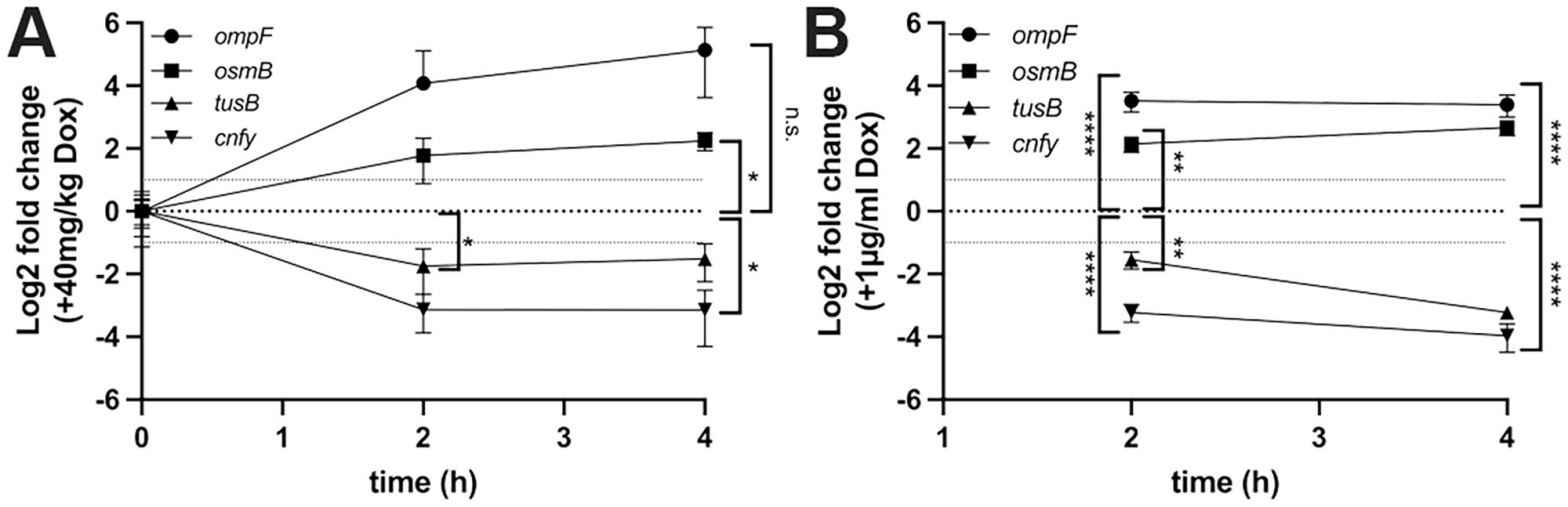
*ompF*, *osmB*, *CNFy*, and *tusB* are differentially regulated in response to doxycycline *in vivo*. **(A)** C57BL/6 mice were infected with WT *Y*. *pseudotuberculosis*, infection proceeded for 48h (hours, h), and mice were injected intraperitoneally with a single dose of 40mg/kg Dox. At the indicated timepoints spleens were harvested, and samples were enriched for bacterial RNA prior to qRT-PCR. Time: h post-treatment, 0h represents 48h post-inoculation (p.i.), the time of treatment. Log2 fold change is relative to 0h. Horizontal dotted lines depict average values for 0h (0log2), and 2-fold changes. Data represents 4 biological replicates/group. **(B)** Cultures of WT *Y*. *pseudotuberculosis* were incubated for 4h in the presence or absence of 1μg/ml Dox, bacterial RNA was isolated and transcripts were detected by qRT-PCR. Log2 fold change values were calculated relative to untreated cells, horizontal dotted lines depict average values for untreated cells (0log2), and 2-fold changes. Data represents four biological replicates. Statistics: (**A)** Two-way ANOVA with Bonferroni’s multiple comparison test, comparisons made relative to 48h p.i./time 0h timepoint. **(B)** Two-way ANOVA with Tukey’s multiple comparison test, comparisons made relative to untreated cells using relative expression values. ****p < .0001, ***p < .001, **p < .01, *p < .05, n.s.: not significant.

Many studies have characterized how bacteria respond to antibiotics during growth in bacteriological media, however, few studies have identified changes that occur within the bacterial population during antibiotic treatment within host tissues. To determine if the gene expression changes we observed in culture are relevant in our mouse model of systemic infection, we infected C57BL/6 mice intravenously with WT *Y*. *pseudotuberculosis*, allowed infection to proceed 48h, and delivered a single injection of 40mg/kg Dox intraperitoneally [18]. Spleens were isolated at 48h (0h, time of Dox administration), or at 2h or 4h post-treatment. RNA was stabilized, isolated, mammalian mRNAs, tRNAs, and rRNAs were depleted to enrich for bacterial RNA, and bacterial transcript levels were detected by qRT-PCR. All 4 genes were differentially regulated in the host environment and the fold change in transcript levels was more dramatic within host tissues than seen with 0.1μg/ml Dox exposure (Fig 2A). *osmB* and *cnfy* had significant changes in transcript levels after 4h treatment, compared to the time of Dox administration (0h) (Fig 2A). For *tusB*, transcripts were significantly lower at 2h post-treatment, but not at 4h. Although there was high upregulation of *ompF* transcript levels, this was not statistically higher than baseline (0h), possibly due to some variability across samples.

Based on our previous study, we believe bacteria are exposed to 0.1–1μg/ml Dox within the mouse spleen in this treatment model, and concentrations may reach as high as 4μg/ml [18,20,39]. To determine if these 4 genes are also differentially regulated in response to 1μg/ml Dox in culture, we performed additional *in vitro* experiments and isolated RNA following 2h and 4h exposure to 1μg/ml Dox to quantify transcript levels by qRT-PCR. All 4 genes were differentially regulated at 2h post-treatment relative to untreated cells, and in contrast with 0.1μg/ml treatment, this differential regulation was maintained 4h post-treatment (Fig 2B). The magnitude of change in transcript levels was also much higher with 1μg/ml compared to 0.1μg/ml Dox, and was between an 8-16-fold change at 4h post-treatment with 1μg/ml Dox (Fig 2B), compared to a maximum 2-fold change with 0.1μg/ml Dox (Fig 1B). The change in transcript levels within the mouse spleen fell in the middle of these two treatment condition values (Fig 2A), consistent with a range of exposure between 0.1–1μg/ml Dox in the spleen.

OmpF is a well described bacterial porin, which is localized in the outer membrane of Gram-negative bacteria, and plays a major role in antibiotic diffusion into the cell [24,36,38]. Interestingly, mutations in *ompF* that either reduce functionality or downregulate expression of the gene have been strongly associated with resistance to β-lactams [40,41]. Our results instead suggest that upregulation of *ompF* could be protective during antibiotic exposure in *Yersinia*. *osmB* encodes for an outer membrane lipoprotein, and its expression is regulated by changes in osmolarity in *E*. *coli* [42,43]. Expression of *osmB* is also regulated by the alternative sigma factor, RpoS, and increases in stationary phase *E*. *coli* [44,45], but *osmB* has not been previously associated with antibiotic susceptibility. However, increased expression of *osmB* in stationary phase could suggest some association of this gene product with slowed growth. TusB functions with TusC and TusD in the sulfur-relay system, which has been associated with translational fidelity, through proper incorporation of glutamic acid, glutamine and lysine into growing polypeptide chains by stabilizing the wobble position of tRNAs [33]. TusB has not been previously associated with antibiotic susceptibility or tolerance. CNFy has been previously associated with persistence in *Y*. *pseudotuberculosis* [28], and for that reason, it will not be a focus in the following figures.

### *ompF* mutants have slightly impaired growth in the presence of doxycycline

The antibiotic susceptibility of a bacterial strain is assessed by several well-defined measurements. These include the minimum inhibitory concentration (MIC): the concentration required for complete bacterial growth inhibition during culture; the minimum bactericidal concentration (MBC): the concentration required for a certain amount of bacterial killing over a defined time period, usually 90% killing or higher; and the more recently described minimum duration for killing (MDK): the amount of time required to kill a bacterial population during exposure to inhibitory levels of drug, typically used to assess the antibiotic tolerance of a bacterial strain [4,5,46–48]. Using this framework, we set-up three sets of *in vitro* assays to assess the antibiotic susceptibility of our WT and mutant strains.

**Fig 3:**
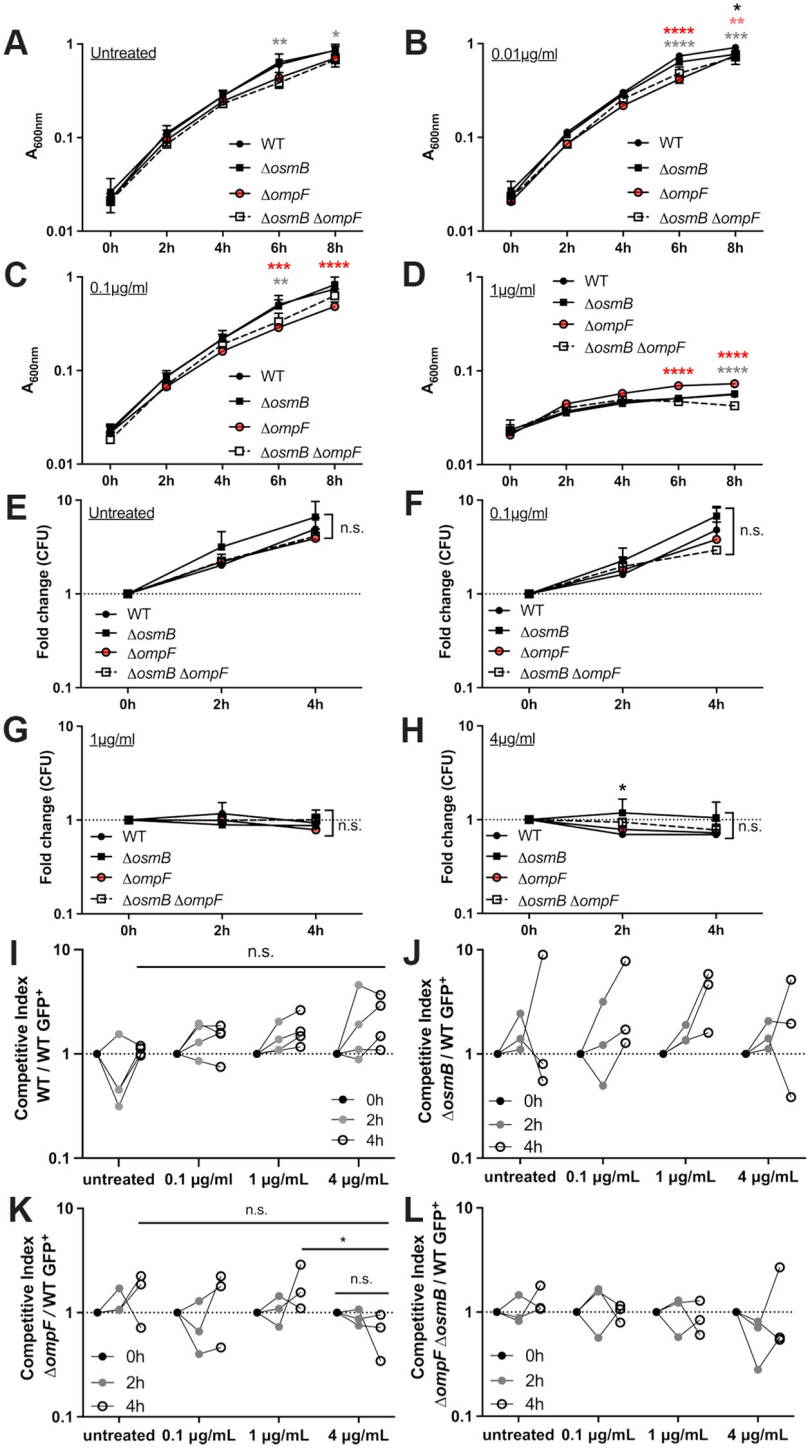
*ompF* mutants have slightly impaired growth in the presence of doxycycline. Exponential phase cultures of the WT and mutant strains (Δ*osmB*, Δ*ompF*, Δ*osmB* Δ*ompF*) were treated with the indicated concentrations of Dox to assess **(A-D)** growth inhibition, **(E-H)** changes in viable counts, and **(I-L)** competitive survival. **(A-D)** Strains were incubated with the indicated concentrations of Dox and growth inhibition was assessed based on absorbance (A_600nm_) at the indicated timepoints (time: hours, h). Data represents the mean and standard deviation of three biological replicates. **(E-H)** Strains were incubated with the indicated concentrations of Dox and viable counts were determined by quantifying CFUs. Fold change in CFUs is shown relative to time 0h. Dotted line: a value of 1, no change relative to 0h. Data represents the mean and standard deviation of three biological replicates. **(I-L)** Competitive survival in the presence of the indicated concentrations of Dox; WT, single and double mutants were tested alongside the WT GFP^+^ strain. Competitive index (CI): CFUs of the test strain/WT GFP^+^ divided by the ratio of test strain /WT GFP^+^ in the culture at time 0h. Values above 1 indicate the mutant preferentially survives, values less than 1 indicate the WT GFP^+^ preferentially survives. Dotted line: value of 1, equal fitness. Dots: biological replicates, lines connect biological replicates sampled across the timepoints, 3–4 biological replicates shown. Statistics: Two-way ANOVA with Tukey’s multiple comparison test, **(A-H)**: comparisons made relative to the WT strain; **(I, K)**: comparisons made between 4h treatment CI values. ****p < .0001, ***p < .001, **p < .01, *p < .05, n.s.: not significant.

To determine if increased expression of *osmB* or *ompF* promotes antibiotic tolerance, we generated single deletion mutants that lack either gene product (Δ*osmB*, Δ*ompF*), and a double deletion mutant strain that lacks both genes (Δ*osmB* Δ*ompF*). We first wanted to test the antibiotic susceptibility of the mutant strains by performing growth curves to determine if the MIC was significantly altered by a loss of these gene products. The MIC should only be altered by the presence or absence of genes required for antibiotic resistance, while gene products contributing to tolerance should not impact the MIC of the strain [4]. Since the WT MIC is 1μg/ml [18], and we would expect mutants to show no change or increased sensitivity, the highest dose used here was 1μg/ml. Under untreated conditions, we found the Δ*osmB* Δ*ompF* strain was already growing slightly slower than the WT strain (Fig 3A). This strain was also growing slower under treated conditions (0.01–1μg/ml) (Fig 3B–3D), suggesting growth rates may be slowed in this strain. Interestingly, the Δ*ompF* strain did appear slightly more sensitive to Dox when compared to the WT strain (0.01–0.1μg/ml) (Fig 3B and 3C). During exposure to inhibitory concentrations (1μg/ml) of Dox, the absorbance of the Δ*ompF* strain was higher than WT, albeit significantly inhibited. Given the growth inhibition of Δ*ompF*, we would have expected the Δ*osmB* Δ*ompF* strain to have similar growth inhibition, however, these results were the first indication that the *osmB* deletion may partially rescue the Δ*ompF* phenotype. These slight changes in growth of the mutant strains were not sufficient to alter their MICs. To confirm these slight changes in growth were due to loss of *ompF*, we attempted to generate an *ompF* rescued strain. However, we were only able to generate a chromosomally-rescued *ompF* in the Δ*osmB* Δ*ompF* background with *osmB* deleted, and it remains unclear why Δ*ompF* could not be rescued directly. These strains (Δ*osmB ompF*^*+*^, *osmB*^*+*^ Δ*ompF*, *osmB*^*+*^ *ompF*^*+*^) showed the expected trends in growth, where Δ*osmB ompF*^*+*^ grew slightly better and *osmB*^*+*^ Δ*ompF* grew slightly worse than the fully rescued strain (*osmB*^*+*^ *ompF*^*+*^), but these absorbance values were not statistically different (S2A Fig).

We then tested the viability of the WT and mutant strains during exposure to heightened, inhibitory levels of Dox (0.1–4μg/ml). The highest concentration used here was 4μg/ml, within the range of peak concentrations in serum in the mouse model (2–10μg/ml) [20,49,50]. We exposed exponential phase cells to Dox for 4h, and plated cells at the point of adding Dox (0h), 2h after exposure, and 4h after exposure to determine whether bacteria continued to grow, were growth inhibited, or lost viability during the exposure. Each strain was grown in a separate test tube with rotation, and we calculated the fold change in CFUs relative to the 0h timepoint. Strains continued to grow under untreated conditions and during exposure to 0.1μg/ml Dox (Fig 3E and 3F), while all strains were growth-inhibited during exposure to 1–4μg/ml Dox (Fig 3G and 3H). There were not significant differences in the viability of the strains, with the exception of the Δ*osmB* strain, which had significantly more CFUs than WT at 2h treatment with 4μg/ml. These data also suggest there is not a detectable difference in the MIC of the mutant strains relative to the WT, and again indicate the MIC for all strains is 1μg/ml. Because bacterial killing was not observed, the MBC or MDK cannot be determined based on these results.

We then tested *in vitro* Dox sensitivity in one additional assay, designed to test competitive survival of two co-cultured strains. The WT strain was transformed with a plasmid that contains a chloramphenicol resistance cassette and also constitutive GFP signal [51,52]. Mutants lacked an antibiotic resistance cassette, allowing us to identify the abundance of each strain by plating on selective and non-selective media. Strains were grown to exponential phase then added to individual wells of a 96-well plate. Mutant strains were tested for competitive survival during co-culture with the WT GFP^+^ strain, and single strain controls were also included to assess growth and survival of individual strains during Dox exposure in the plate, to ensure this mirrored the viability results. Competitive index values were calculated based on the ratio of mutant/WT GFP^+^ CFUs at the indicated timepoints after treatment relative to the ratio of mutant/WT GFP^+^ CFUs in the well at 0h, when Dox was added. Competition between an unmarked WT and WT GFP^+^ was included as a control, and showed a WT unmarked strain outcompetes WT GFP^+^ at high Dox concentrations, although this was not statistically significant (Fig 3I). Data comparing survival of the WT and Δ*osmB* strain suggested that the Δ*osmB* may have a slight fitness advantage relative to the WT strain, and showed no increased Dox sensitivity (Fig 3J). Consistent with the slightly impaired growth of the Δ*ompF* strain (Fig 3B and 3C), Δ*ompF* showed decreased relative survival during exposure to 4μg/ml Dox, especially when compared the WT unmarked strain (Fig 3I), suggesting this strain does have increased Dox sensitivity (Fig 3K). Interestingly, the Δ*osmB* Δ*ompF* strain had very similar survival compared to the WT strain (Fig 3L), suggesting this strain does not have Dox sensitivity and that the *osmB* deletion may partially rescue the Δ*ompF* phenotype. To confirm these changes in competitive survival were due to loss of *ompF*, we again utilized the chromosomally-rescued mutants in the Δ*osmB* Δ*ompF* strain background. The *osmB*^*+*^ Δ*ompF* strain showed slightly more sensitivity to 4μg/ml than 1μg/ml Dox, similar to Δ*ompF*, although this was not statistically significant (S2B Fig). Without a significant phenotype here, and with high levels of variability in the assay, it was difficult to assess rescue with the *osmB*^*+*^ *ompF*^*+*^ strain; however, the average CI value of 1 suggests *osmB*^*+*^ *ompF*^*+*^ competes similarly to WT GFP^+^. Collectively, these data suggest the Δ*ompF* strain has increased Dox sensitivity, however the *osmB* deletion has no detectable impact on Dox sensitivity, and actually seems to improve the fitness of the strain.

### Δ*osmB* has increased *in vivo* fitness relative to the WT strain, Δ*ompF* does not appear significantly more susceptible to doxycycline *in vivo*

To determine if the Δ*osmB* or Δ*ompF* strains had increased doxycycline (Dox) sensitivity *in vivo*, we tested their survival in our mouse model of infection during exposure to a single dose of Dox. Because the *in vitro* phenotypes were subtle, we decided to test the survival of mutant strains relative to the WT in a co-infection experiment, essentially mirroring the competitive survival experiments we had set-up in culture (Fig 3J and 3K). Mutant strains used for infection contained a *yopE*::*mCherry* construct that would allow us to detect mutant cells with mCherry fluorescence [52,53]. The Δ*osmB* Δ*ompF* strain was not tested here since it showed no difference in antibiotic susceptibility relative to the WT strain (Fig 3L).

**Fig 4:**
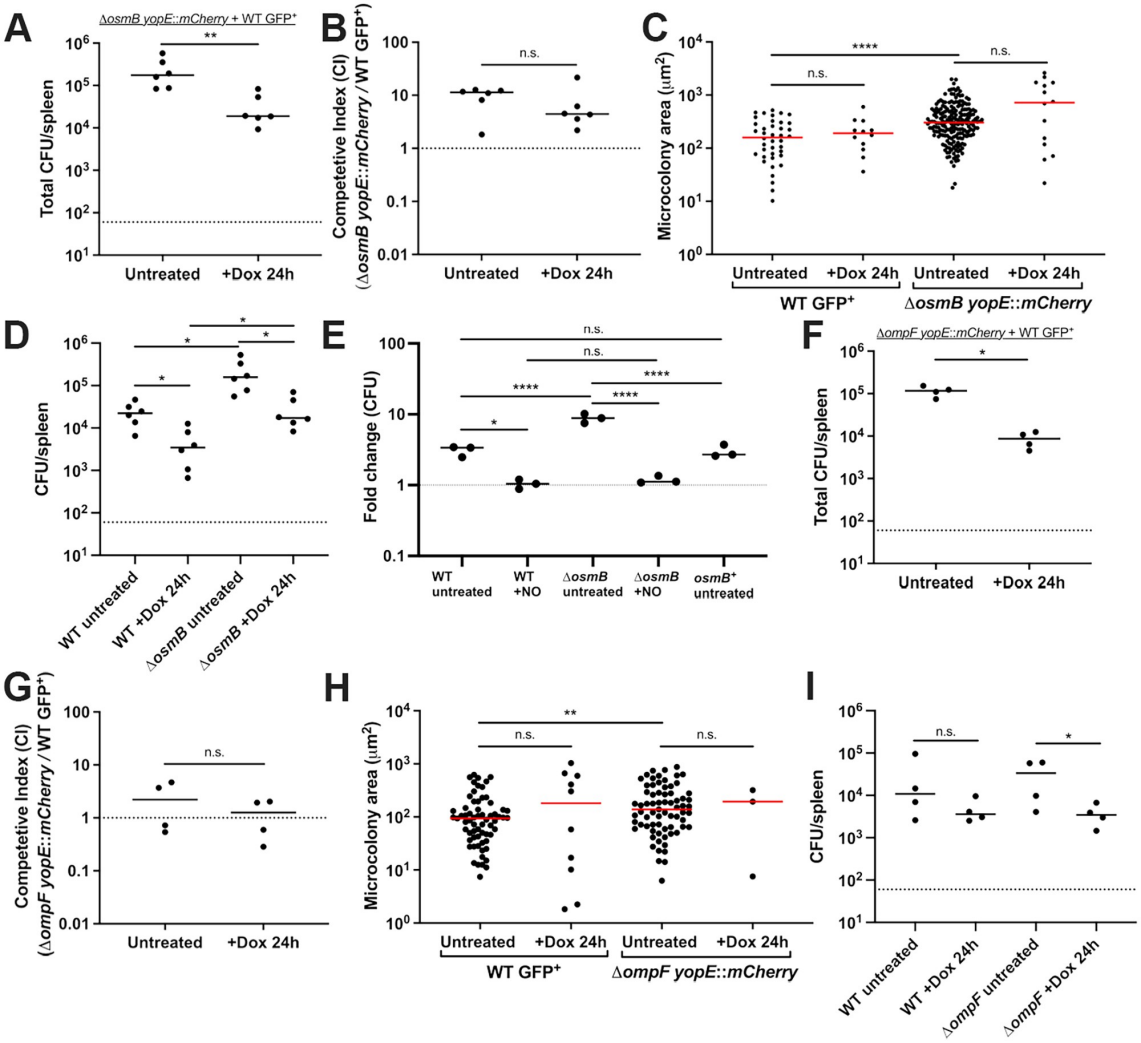
Δ*osmB* has increased *in vivo* fitness relative to the WT strain, Δ*ompF* does not appear significantly more susceptible to doxycycline *in vivo*. C57BL/6 mice were infected with equal numbers of WT and mutant *Y*. *pseudotuberculosis*, infection proceeded for 48h (hours, h), and mice were injected intraperitoneally with a single dose of Dox or left untreated. Spleens were harvested 24h later to quantify CFUs and microcolony areas. **(A)** Co-infection with Δ*osmB yopE*::*mCherry* + WT GFP^+^. Total CFU/spleen for treated mice (+Dox 24h) compared to untreated mice at the same timepoint. Dots: individual mice. **(B)** Competitive index: CFUs of the mutant/WT in the spleen divided by the ratio of mutant/WT in the inoculum. Values above 1: mutant is more fit. Dotted line: value of 1, equal fitness. Dots: individual mice. **(C)** Microcolony areas were quantified for individual strains within the harvested spleens (6 untreated mice and 6 treated mice). 20–62 microcolonies were quantified in one section for untreated mice, and 1–13 microcolonies were quantified in one section for treated mice. **(D)** CFU/spleen of each strain in the Δ*osmB* + WT competition experiments. **(E)** Fold change in bacterial growth after 4h culture in the presence (+NO) or absence (untreated) of the NO donor compound, DETA-NONOate (2.5mM). WT, Δ*osmB*, and *osmB*^*+*^ (*osmB* rescued strain) are compared. Fold change relative to time 0, dotted line represents time 0 values. (**F**) Co-infection with Δ*ompF yopE*::*mCherry* + WT GFP^+^. Total CFU/spleen for treated mice (+Dox 24h) compared to untreated mice at the same timepoint. Dots: individual mice. **(G)** Competitive index: CFUs of the mutant/WT in the spleen divided by the ratio of mutant/WT in the inoculum. Values above 1: mutant is more fit. Dotted line: value of 1, equal fitness. Dots: individual mice. **(H)** Microcolony areas were quantified for individual strains within the harvested spleens (4 untreated mice and 4 treated mice). 14–72 microcolonies were quantified in one section for untreated mice, and 1–7 microcolonies were quantified in one section for treated mice. **(I)** CFU/spleen of each strain in the Δ*ompF* + WT competition experiments. All mouse experiments were carried out on two independent days. Statistics: **(A, B, C, F, G, H)** Mann-Whitney; **(E)** One-way ANOVA with Tukey’s post-test; **(D, I)** Kruskal-Wallis one-way ANOVA with Dunn’s post-test. ****p < .0001, **p < .01, *p < .05, n.s.: not significant.

Equal amounts of the WT and mutant strain were mixed, mice were inoculated intravenously, and 48h post-inoculation mice received a single dose of Dox or were left untreated. 24h after Dox injection (+Dox 24h), spleens were harvested to quantify CFUs and examine microcolony areas by fluorescence microscopy. The WT strain was marked with a constitutive GFP plasmid and chloramphenicol resistance, and the mutants contained *yopE*::*mCherry* to mark all mutant cells with mCherry signal, as described above. Total CFU/spleen were first quantified to ensure Dox treatment significantly decreased bacterial CFUs, then the numbers and microcolony areas of each strain were quantified. Although the Dox treatment significantly decreased CFUs (Fig 4A), the Δ*osmB* strain outcompeted the WT strain in the presence and absence of Dox (Fig 4B), again showing the Δ*osmB* strain does not have increased Dox sensitivity, and actually has a fitness advantage relative to WT. Consistent with this, the microcolony areas of the Δ*osmB* strain appeared slightly larger than WT, although this was not statistically significant (Fig 4C). The number of microcolonies decreased with treatment for both the WT and Δ*osmB* strains, but microcolonies did not significantly decrease in size after treatment for either strain (Fig 4C). We were expecting to see decreased microcolony areas after treatment, but that may have been difficult to assess without comparing to the areas at the time of injection (48h) [18]. We then quantified the CFUs of each strain before and after treatment within the same tissues. There were more Δ*osmB* CFUs prior to and after treatment, and both WT and Δ*osmB* CFUs significantly dropped in response to treatment (Fig 4D).

To determine if the increased growth of the Δ*osmB* strain in the mouse model could be due to reduced sensitivity to nitric oxide (NO), we completed *in vitro* experiments with the NO donor compound, DETA-NONOate. WT, Δ*osmB*, and the *osmB*^*+*^ (*osmB* rescue) strains were grown in the absence and presence of 2.5mM DETA-NONOate for 4h, and sensitivity was assessed based on CFUs. As shown in previous studies [18,51,54], this concentration inhibits growth of the WT strain, and we observed similar levels of growth inhibition with the Δ*osmB* strain, suggesting increased growth *in vivo* is not due to altered NO sensitivity (Fig 4E). We also observed a clear increase in growth of the Δ*osmB* strain relative to WT under untreated conditions, which was rescued in the *osmB*^*+*^ strain (Fig 4E). These data together show that the loss of *osmB* improves growth of *Y*. *pseudotuberculosis*, both *in vitro* and *in vivo*.

OsmB contributes to membrane repair after exposure of stationary phase *E*. *coli* to high pressure conditions [55]. Because *osmB* is also expressed in *E*. *coli* under high osmolarity conditions [43–45], we hypothesized OsmB would be required for *Y*. *pseudotuberculosis* survival under high osmolarity conditions. To better understand the role of OsmB, we cultured exponential and stationary phase WT, Δ*osmB*, and the *osmB*^*+*^ (*osmB* rescue) strains under high osmolarity conditions (1M sorbitol, 0.5M and 1M NaCl), which have been shown to induce *osmB* expression [44]. We found all strains were similarly sensitive to high osmolarity conditions in both stationary and exponential phase suggesting *osmB* is not required (S3A and S3B Fig). These results are consistent with redundancy in the pathways that protect cells from outer membrane stress, and suggest additional lipoproteins are sufficient to protect cells when *osmB* is deleted.

During co-infection with the WT and Δ*ompF* strains, we confirmed Dox treatment significantly decreased CFUs (Fig 4F). However, the WT and Δ*ompF* strains were equally fit in the absence and presence of Dox, hence while the Δ*ompF* strain may have slight increased susceptibility to Dox *in vitro*, this wasn’t sufficient to impact survival of the strain within mouse tissues (Fig 4G). The microcolony areas of the WT and Δ*ompF* strains were similar, although the Δ*ompF* microcolonies were slightly larger than WT in untreated mice, but the areas were not significantly altered by treatment (Fig 4H). There were few Δ*ompF* microcolonies remaining within host tissues after treatment, which may suggest increased susceptibility of the strain. However, the number of WT microcolonies in the same spleens also decreased (Fig 4H). The CFUs of the WT and Δ*ompF* strain were similar before and after treatment, however the decrease in Δ*ompF* CFUs after treatment was more pronounced, which could indicate an increase in Δ*ompF* Dox sensitivity *in vivo* (Fig 4I).

### Δ*ompF* has altered doxycycline permeability

Increased expression of the OmpF porin in response to Dox was unexpected, since OmpF expression could increase the diffusion of antibiotic into bacterial cells [36,40,41]. We hypothesized that Dox may use a different outer membrane protein for entry into *Y*. *pseudotuberculosis*, and that increased expression of *ompF* may promote antibiotic diffusion back out of the cell. This would lead to increased levels of Dox accumulation in the Δ*ompF* strain, where Dox could enter the cell, but not diffuse out. Similarly, we hypothesized that OsmB lipoprotein could impact membrane permeability, and sought to determine if there was also differential Dox accumulation in Δ*osmB* cells. However, the limited impact of OsmB on Dox sensitivity meant that any change in Dox accumulation in the Δ*osmB* strain would likely be minimal.

**Fig 5:**
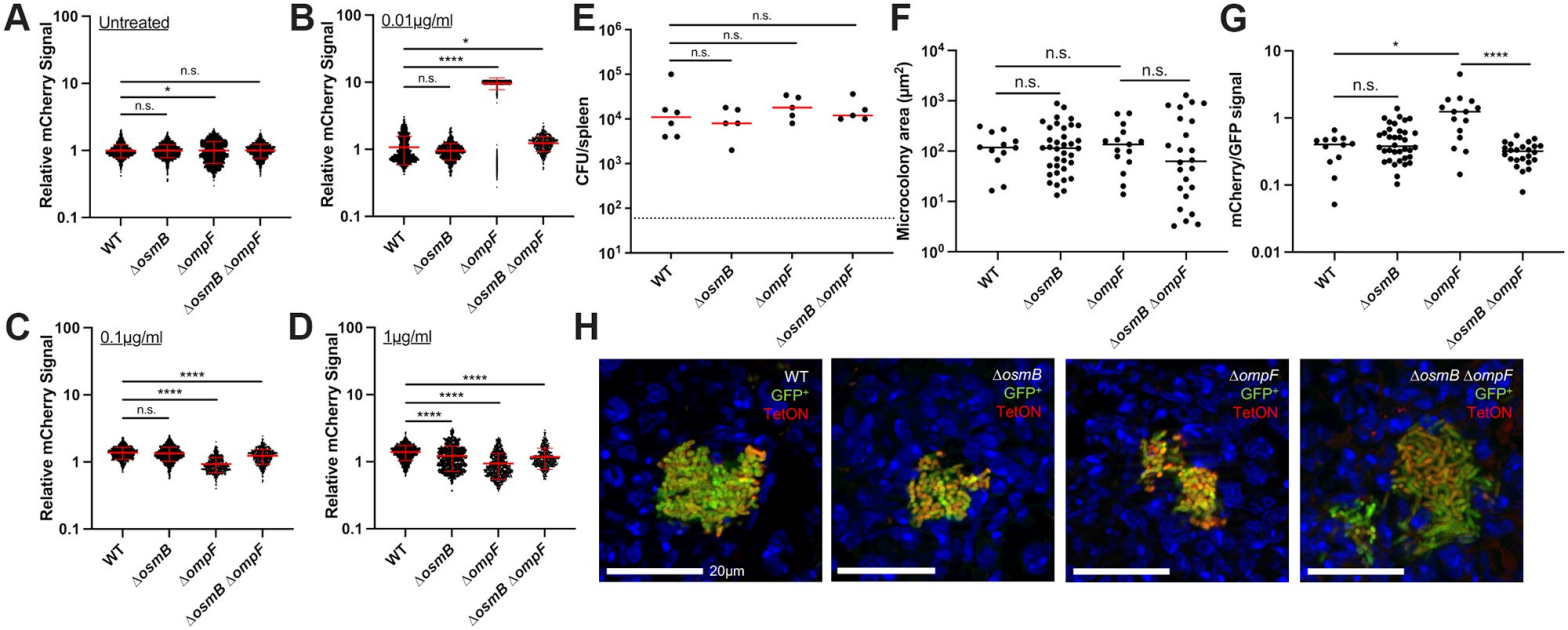
Δ*ompF* has altered doxycycline permeability. **(A-D)** WT and deletion strains (Δ*osmB*, Δ*ompF*, Δ*osmB* Δ*ompF*) transformed with the constitutive GFP plasmid and TetON reporter plasmid were incubated with the indicated concentrations of Dox for 4h. mCherry fluorescence was detected within individual cells by fluorescence microscopy. Relative mCherry was calculated by normalizing to the WT untreated average cell value. Data represents the mean and standard deviation of three biological replicates. **(E)** C57BL/6 mice were infected with WT or mutant strains (GFP^+^ TetON). Infections proceeded 48h, and mice were injected intraperitoneally with a single dose of Dox. Spleens were harvested 24h after treatment to **(E)** quantify CFUs, **(F)** quantify microcolony areas, and **(G)** quantify reporter signal by fluorescence microscopy. Relative reporter signal was quantified by dividing the mean mCherry (TetON) signal by the mean GFP signal from each microcolony. 5 mice were quantified/strain, and 1–15 microcolonies were quantified in one section for each mouse. **(H)** Representative images, scale bars: 20μm. All mouse experiments were carried out on two independent days. Statistics: Kruskal-Wallis one-way ANOVA with Dunn’s post-test. ****p < .0001, *p < .05, n.s.: not significant.

To determine if Dox differentially accumulates in mutant strains, we utilized a TetON reporter system to detect intracellular Dox concentrations. In this reporter system, increasing concentrations of Dox results in heightened levels of mCherry signal, until concentrations are sufficient to inhibit mCherry translation [18]. This results in a bell curve, or normal distribution, of mCherry expression. The WT and deletion strains (Δ*osmB*, Δ*ompF*, Δ*osmB* Δ*ompF*) were first transformed with the constitutive GFP^+^ then transformed with the TetON reporter plasmid. The GFP signal was used here to select individual cells. Strains were exposed to increasing concentrations of Dox in culture, and we quantified mCherry fluorescence within individual cells by fluorescence microscopy. In the absence of treatment, background mCherry fluorescence was similar in all strains, but slightly higher in Δ*ompF* (Fig 5A). With low concentrations of Dox (0.01μg/ml), we detected high levels of fluorescence in the Δ*ompF* strain, consistent with increased antibiotic accumulation (Fig 5B). Interestingly, the Δ*osmB* Δ*ompF* had slightly increased fluorescence compared to the WT strain, but much lower fluorescence than Δ*ompF*, again indicating deletion of *osmB* may have partially rescued the *ompF* phenotype (Fig 5B). Consistent with increased antibiotic accumulation in Δ*ompF*, exposure to 0.1μg/ml Dox caused fluorescence to drop below WT levels, suggesting this relatively low concentration was accumulating to sufficient levels to inhibit mCherry translation (Fig 5C). Exposure to 1μg/ml Dox resulted in decreased mCherry fluorescence in all mutants relative to WT, suggesting they may all have some level of increased antibiotic accumulation (Fig 5D). Throughout these *in vitro* experiments we saw the most dramatic changes with Δ*ompF*, providing evidence this gene product impacts antibiotic accumulation within the cell. The impact of *osmB* was subtle, while the Δ*osmB* Δ*ompF* strain showed an intermediate phenotype; this will be discussed in more detail in the Discussion.

To determine if differential antibiotic accumulation occurs in mutant strains during infection, we performed single strain infections with WT, Δ*osmB*, Δ*ompF*, or Δ*osmB* Δ*ompF* GFP^+^ TetON strains, allowed infection to proceed 48h, injected intraperitoneally with Dox, and harvested spleens 24h after treatment to quantify CFUs, microcolony areas, and detect reporter signal by fluorescence microscopy. The constitutive GFP signal was used to assess differential TetON (mCherry) reporter expression across microcolonies [18]. The CFUs and microcolony areas for each strain were similar, consistent with the previous findings in Fig 4B and 4E, indicating these strains are not significantly more sensitive to Dox than WT (Fig 5E and 5F). The relative Dox exposure was then quantified by dividing the mean mCherry signal by the mean GFP signal for each microcolony. Δ*ompF* microcolonies had significantly higher reporter signal relative to the other strains, suggesting that heightened Dox accumulation also occurs within this mutant during infection (Fig 5G and 5H). It is important to note that while Δ*ompF* may accumulate more Dox, this is not sufficient to significantly impact bacterial survival (Fig 5E) or microcolony area (Fig 5F) when comparing across single strain infections, indicating the impact of OmpF on antibiotic susceptibility remains subtle. We again observed fewer microcolonies within Δ*ompF* infected tissues; it required sectioning deeper into tissues to find bacterial centers, indicating the mutant strain does not retain as many microcolonies after treatment.

To show that altered antibiotic accumulation is due to loss of *ompF*, we utilized the chromosomally-rescued *ompF* strains in the Δ*osmB* Δ*ompF* background. We performed *in vitro* antibiotic accumulation experiments in strains that contained the TetON plasmid (Δ*osmB* Δ*ompF*, Δ*osmB ompF*^*+*^, *osmB*^*+*^ Δ*ompF*, *osmB*^*+*^ *ompF*^*+*^, WT), and quantified mCherry fluorescence within individual cells by microscopy after 4h exposure to 0.01 or 0.1 μg/ml Dox. We expected to see a clear peak of mCherry signal in the mutant strains at 0.01μg/ml, which we did not observe (S4 Fig). At 0.01μg/ml Dox, the Δ*osmB* Δ*ompF* strain had lower fluorescence than the other strains, which was not consistent with the GFP^+^ Δ*osmB* Δ*ompF* strain in Fig 5, and rescues of this strain suggested both *osmB* and *ompF* promote antibiotic accumulation based on heightened signal (S4 Fig). All strains increased in mCherry fluorescence with 0.1μg/ml Dox, and all mutant strains had lower signal than WT. This suggested both *ompF* and *osmB* mutants had altered Dox uptake, but this was not restored to WT levels in the fully rescued strain (*osmB*^*+*^ *ompF*^*+*^) (S4 Fig). It is important to note these strains were independently-derived from unmarked Δ*osmB* Δ*ompF*, and these results suggest the strains may have acquired additional genetic modifications that both impacted our ability to rescue *ompF*, and altered the phenotypes of rescued strains.

### *tusB* overexpression promotes *Y*. *pseudotuberculosis* doxycycline sensitivity *in vitro*

**Fig 6:**
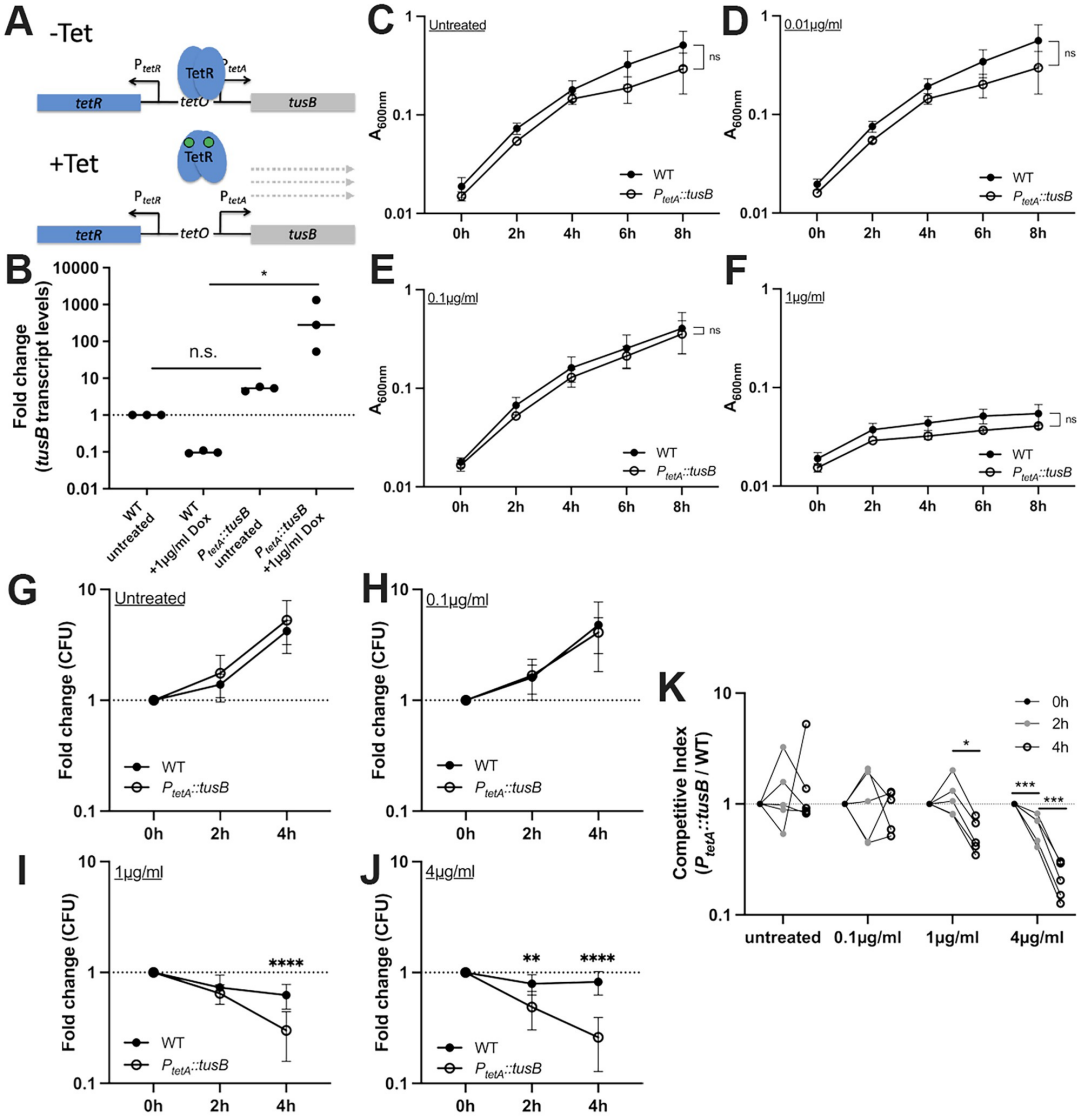
*tusB* overexpression promotes *Y*. *pseudotuberculosis* doxycycline sensitivity *in vitro*. **(A)** Schematic of *tusB* overexpression strain. *tusB* expression is repressed by TetR in the absence of tetracyclines (-Tet). In the presence of antibiotics (+Tet), TetR repression is relieved, and *tusB* is expressed. **(B)** qRTPCR was used to detect *tusB* transcript levels in WT and *P*_*tetA*_::*tusB* strains in the absence and presence of 1μg/ml Dox (4h treatment). Fold change shown relative to *tusB* transcript levels in untreated WT cells (normalized to a value of one, dotted line), median values depicted, three biological replicates are shown. Exponential phase cultures of the WT and *tusB* overexpression strain (*P*_*tetA*_::*tusB*) were treated with the indicated concentrations of Dox to assess **(C-F)** growth inhibition, **(G-J)** changes in viable counts, and **(K)** competitive survival. **(C-F)** Strains were incubated with the indicated concentrations of Dox and growth inhibition was assessed based on absorbance (A_600nm_) at the indicated timepoints (time: hours, h). Data represents the mean and standard deviation of five biological replicates. **(G-J)** Strains were incubated with the indicated concentrations of Dox and viable counts were determined by quantifying CFUs. Fold change in CFUs is shown relative to time 0h. Dotted line: a value of 1, no change relative to 0h. Data represents the mean and standard deviation of ten biological replicates. **(K)** Competitive survival in the presence of the indicated concentrations of Dox. Competitive index: CFUs of the *P*_*tetA*_::*tusB* /WT divided by the ratio of *P*_*tetA*_::*tusB* /WT in the culture at time 0h. Values above 1 indicate the *tusB* strain preferentially survives, values less than 1 indicate the WT preferentially survives. Dotted line: value of 1, equal fitness. Dots: biological replicates, lines connect biological replicates sampled across the timepoints, five biological replicates shown. Statistics: **(B)** Friedman paired one-way ANOVA with Dunn’s post-test, **(C-K)** Two-way ANOVA with Bonferroni’s multiple comparison test, **(C-J)**: comparisons made relative to the WT strain; **(K)**: comparisons made between timepoints at the same Dox concentration. ****p < .0001, ***p < .001, **p < .01, *p < .05, n.s.: not significant.

tRNA 2-thiouridine synthesizing protein B (*tusB*) is a protein that functions as a heterohexamer with TusC and TusD in the sulfur-relay system, responsible for the post-transcriptional 2-thiolation of the uracil 34 at the wobble position [33,34]. This modification is necessary for the identification of purines, specifically promoting proper translation and incorporation of glutamic acid, glutamine and lysine into growing polypeptide chains [33]. Our RNA-seq results showed that *tusB* is significantly downregulated in response to Dox, thus, we chose to generate a tet-inducible (TetON) *tusB* overexpression strain (*P*_*tetA*_::*tusB*), to specifically induce *tusB* expression in the presence of Dox and prevent downregulation [18] (Fig 6A). We used qRTPCR to detect *tusB* transcript levels in WT and *P*_*tetA*_::*tusB* strains under untreated conditions and during 4h exposure to 1μg/ml Dox, and confirmed *P*_*tetA*_::*tusB* results in *tusB* overexpression in the presence of Dox (Fig 6B). It is important to note the *P*_*tetA*_::*tusB* strain has heightened levels of *tusB* prior to Dox exposure as well, although these levels are not statistically different from WT cells (Fig 6B). To determine if the Dox MIC was altered by increased expression of *tusB*, we performed growth curves with increasing levels of Dox. Overexpression of *tusB* did not alter bacterial growth kinetics or the MIC of the strain, and we observed similar absorbance levels for the WT and *P*_*tetA*_::*tusB* strains under untreated and treated conditions (Fig 6C–6F).

We then tested the viability of the WT and *P*_*tetA*_::*tusB* strains during exposure to heightened, inhibitory levels of Dox (0.1–4μg/ml). As described above, we exposed exponential phase cells to Dox for 4h, and plated cells at the point of adding Dox (0h), 2h, and 4h after exposure to quantify the number of viable cells during the exposure. Strains were grown in separate test tubes with rotation, and we calculated the fold change in CFUs relative to the 0h timepoint. As seen with the *osmB* and *ompF* mutants, strains continued to grow under untreated conditions and during exposure to 0.1μg/ml Dox (Fig 6G and 6H). However, there was a significant drop in the number of *P*_*tetA*_::*tusB* cells at 4h of treatment with 1μg/ml Dox compared to WT cells (Fig 6I). This phenotype was more pronounced with 4μg/ml Dox, where there was a significant drop in *P*_*tetA*_::*tusB* cells relative to WT after only 2h exposure, and greater loss in viability at 4h post-treatment (Fig 6J). These data strongly support a role for *tusB* downregulation in Dox tolerance, and show that Dox can have bactericidal activity against the *P*_*tetA*_::*tusB* strain. This defines the *P*_*tetA*_::*tusB* strain as susceptible, and the WT strain as tolerant under these conditions.

We also tested the competitive survival of the *P*_*tetA*_::*tusB* strain relative to WT during co-culture. The *P*_*tetA*_::*tusB* construct contains a chloramphenicol resistance cassette, which was used to quantify the CFUs of this strain. An unmarked WT strain was used alongside *P*_*tetA*_::*tusB*. Exponential phase cells were added to individual wells of a 96 well plate, and competitive index values were calculated based on the ratio of *P*_*tetA*_::*tusB* /WT CFUs at the indicated timepoints after treatment relative to the ratio when Dox was added. These data were consistent with the viability count data, and showed the *P*_*tetA*_::*tusB* strain was significantly outcompeted by WT during Dox exposure (Fig 6K). The *P*_*tetA*_::*tusB* strain lost significantly more viable cells than WT during 4h exposure to 1μg/ml Dox, and at both 2h and 4h after exposure to 4μg/ml Dox. Both methods of detecting antibiotic tolerance showed that *tusB* downregulation significantly contributes to antibiotic tolerance, and that reversing this phenotype by expressing *tusB* during Dox treatment results in heightened drug susceptibility. Bacterial killing was observed in the *P*_*tetA*_::*tusB* strain, although we would need to extend experiments longer to accurately determine MBC and MDK values with 90% killing or more stringent cut-offs. However, based on these experiments, approximately 75% of cells were killed with 4μg/ml Dox at 4h, which would mean the MBC_75_ is ~4μg/ml and the MDK_75_ would be ~4h.

### *tusB* expressing cells have increased susceptibility to doxycycline *in vivo*

**Fig 7:**
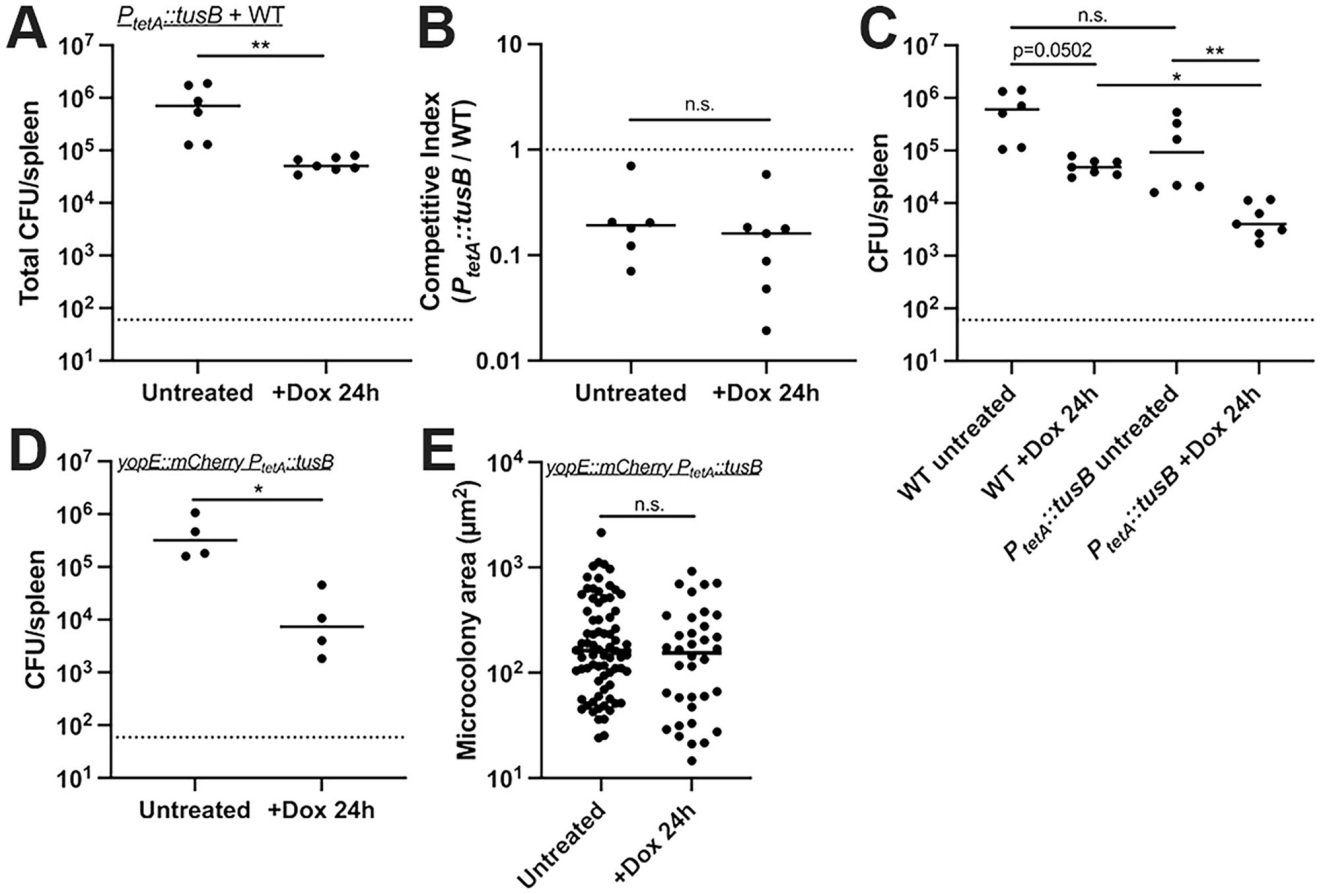
*tusB* expressing cells have increased susceptibility to doxycycline *in vivo*. C57BL/6 mice were infected with equal numbers of the WT and *P*_*tetA*_::*tusB* strains, infection proceeded for 48h, and mice were either injected intraperitoneally with a single dose of Dox or left untreated. 24h later spleens were harvested to quantify CFUs. **(A)** Total CFU/spleen, each dot represents an individual mouse. **(B)** Competitive index values. Dotted line: value of 1; equal fitness between the two strains. Values less than 1 indicate the *tusB* strain is less fit than WT. **(C)** CFU/spleen of each strain in the WT + *P*_*tetA*_::*tusB* competition experiments. **(D)** C57BL/6 mice were infected with the *yopE*::*mCherry P*_*tetA*_::*tusB* strain, infection proceeded for 48h, and mice were either injected intraperitoneally with a single dose of Dox or left untreated. 24h later spleens were harvested to quantify CFUs. **(E)** Microcolony areas of the *yopE*::*mCherry P*_*tetA*_::*tusB* strain were quantified from 4 untreated mice and 4 treated mice, each dot represents an individual microcolony. 6–38 microcolonies were quantified in one section for untreated mice, and 1–19 microcolonies were quantified in one section for treated mice. All mouse experiments were carried out on two independent days, horizonal lines depict median values. Statistics: **(A, B, D, E)** Mann-Whitney; **(C)** Kruskal-Wallis one-way ANOVA with Dunn’s post-test. **p < .01, *p < .05, n.s.: not significant.

To determine if the increased Dox sensitivity of the *P*_*tetA*_::*tusB* strain resulted in impaired survival during Dox treatment in the mouse model of infection, we compared the survival of the WT and *P*_*tetA*_::*tusB* strains during co-infection. Equal amounts of the WT and *P*_*tetA*_::*tusB* strain were mixed, mice were inoculated intravenously, then at 48h post-inoculation mice either received a single dose of doxycycline or were left untreated. At 24h post-treatment, spleens were harvested to quantify CFUs. Enumeration of total CFUs indicated that the Dox was sufficient to significantly decrease CFUs (Fig 7A), however the competitive index values were similar with and without treatment (Fig 7B). Notably, the competitive index values were below one, indicating the *P*_*tetA*_::*tusB* strain was slightly outcompeted by WT in mouse tissues even under untreated conditions, despite very similar fitness in culture. We then quantified the CFUs of each strain before and after treatment within the same tissues. We saw a pronounced reduction in the CFUs of the *P*_*tetA*_::*tusB* strain after treatment and also a reduction in WT CFUs, although this was not statistically significant (p = 0.0502) (Fig 7C). While the WT and *P*_*tetA*_::*tusB* CFUs were similar prior to treatment, there was a significant drop in *P*_*tetA*_::*tusB* CFUs relative to WT +Dox treatment, indicating the *P*_*tetA*_::*tusB* strain does have heightened sensitivity to Dox within mouse tissues (Fig 7C). To determine if this heightened sensitivity also results in smaller microcolony areas after treatment, we transformed WT *Y*. *pseudotuberculosis* containing *yopE*::*mCherry* with the *P*_*tetA*_::*tusB* plasmid and repeated infections with this strain. Mice were inoculated intravenously with the *yopE*::*mCherry P*_*tetA*_::*tusB* strain, at 48h post-inoculation mice received a single dose of Dox or were left untreated, and spleens were harvested 24h post-treatment. The CFUs of the *yopE*::*mCherry P*_*tetA*_::*tusB* strain significantly decreased with treatment (Fig 7D), however this was not reflected by a change in microcolony size (Fig 7E). We did observe there were fewer total microcolonies in treated tissues, as we had also seen with the WT, Δ*osmB*, and Δ*ompF* infections. These results could suggest that larger and smaller microcolonies are equally susceptible to treatment, and may be eliminated or survive at a similar rate.

### *ompF*, *tusB*, and *cnfy* are differentially regulated in response to inhibitory concentrations of chloramphenicol, while *osmB* expression was only altered by doxycycline

**Fig 8:**
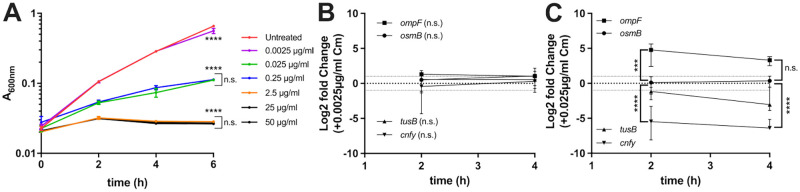
*ompF*, *tusB*, and *cnfy* are differentially regulated in response to inhibitory concentrations of chloramphenicol, while *osmB* expression was only altered by doxycycline. **(A)** WT *Y*. *pseudotuberculosis* was grown in the presence of the indicated concentrations of chloramphenicol (Cm). Growth was measured by absorbance (A_600nm_) at the indicated timepoints (hours, h). Bacteria were grown in the presence or absence of **(B)** 0.0025μg/ml Cm or **(C)** 0.025μg/ml Cm. RNA was isolated after 2h and 4h exposure, and the indicated transcripts were detected by qRT-PCR. Fold change is relative to untreated cells (represented by a dotted line at a value of 1). Statistics: **(A-C)** Two-way ANOVA with Tukey’s multiple comparison test, comparisons made relative to untreated cells, comparisons between treatment conditions where indicated by brackets. In C, statistics represent treated vs. untreated comparisons for *cnfy*, and *ompF* compared to *osmB*, which did not change with treatment. ****p < .0001, ***p < .001, n.s.: not significant.

Many antibiotics target the ribosome to inhibit translation and arrest bacterial growth. To begin to determine if the differential regulation of *osmB*, *ompF*, *tusB* and *cnfy* was a response to doxycycline, or a more general response to ribosomal inhibition, we also performed experiments with chloramphenicol (Cm), which targets the 50S ribosomal subunit by binding to 23S rRNA [56]. Cm was also chosen for these experiments because it can be used to treat *Yersinia* infections [21,23]. We performed growth curves with increasing doses of Cm (0.0025μg/ml-50μg/ml) to determine the sensitivity of WT *Y*. *pseudotuberculosis* to Cm, and define the minimum inhibitory concentration (MIC). All concentrations tested resulted in significant growth inhibition after 6 hours of growth in bacteriological media (Fig 8A). However, the growth curve with 0.0025μg/ml chloramphenicol mirrored untreated growth through 4 hours of growth, suggesting this low dose minimally impacted growth, and was sub-inhibitory. Treatment with 0.025–0.25μg/ml resulted in similar levels of growth inhibition, and treatment with 2.5–50μg/ml resulted in severe growth inhibition, to the point that no growth was detected (Fig 8A). Based on these data, the MIC of our WT *Y*. *pseudotuberculosis* strain to Cm was 2.5μg/ml.

We then compared the levels of *osmB*, *ompF*, *tusB* and *cnfy* transcripts during exposure to the subinhibitory 0.0025μg/ml Cm dose and the inhibitory 0.025μg/ml dose. The subinhibitory 0.0025μg/ml Cm did not significantly change any transcript levels when comparing untreated and treated cells (Fig 8B). Interestingly, the inhibitory 0.025μg/ml Cm dose did not change *osmB* transcript levels, which may suggest that the change in *osmB* transcripts in response to Dox is antibiotic-specific, and not a general response to ribosomal inhibition (Fig 8C). *ompF* was significantly upregulated in response to this concentration of Cm, and *cnfy* was significantly downregulated, suggesting these could be general responses to ribosomal inhibition. *tusB* transcript levels also decreased in response to Cm, although this was not statistically significant. Collectively, these data suggest that transcriptional regulation of *ompF*, *cnfy*, and *tusB* may be a response to ribosomal targeting by antibiotics, while heightened *osmB* expression may be a specific response to doxycycline.

## Discussion

Antibiotic tolerance is associated with a phenotypic change within a bacterial population that results in decreased antibiotic susceptibility. Tolerance is specifically associated with bactericidal antibiotics, however, under conditions where bacteriostatic antibiotics are directly killing cells, the terminology can also be applied to a bacteriostatic antibiotic, such as doxycycline (Dox) [14,16,17]. Here, we sought to determine how bacteria respond to Dox exposure in culture, and determine whether differential gene expression is required for surviving Dox treatment in culture and in our mouse model of *Y*. *pseudotuberculosis* infection. Only four genes were differentially regulated ~2 fold in response to a physiologically-relevant dose of Dox (Fig 1B): *osmB*, *ompF*, *tusB* and *cnfy;* differential gene expression occurred in response to low and high doses of Dox and during antibiotic treatment in our mouse model (Fig 2A and 2B). We then tested the role of *osmB*, *ompF*, and *tusB* differential expression in doxycycline tolerance by generating deletion mutants for the genes with heightened expression (*osmB*, *ompF*), and a *P*_*tetA*_::*tusB* construct to specifically promote heightened *tusB* expression during Dox exposure. Using these strains, we found that *osmB* expression does not significantly contribute to Dox susceptibility, *ompF* expression impacts Dox accumulation within bacterial cells and has a slight contribution to tolerance, while *tusB* downregulation significantly contributes to Dox tolerance. *cnfy* expression has previously been associated with persistence in *Y*. *pseudotuberculosis* [28], and so we chose to focus on *osmB*, *ompF*, and *tusB* in our experiments here. Interestingly, *ompF*, *tusB* and *cnfy* were also differentially regulated in response to chloramphenicol, suggesting these could be general responses to ribosomal inhibition, while *osmB* upregulation may be more specific to Dox (Fig 8C).

Expression of the *osmB* lipoprotein impaired bacterial growth, based on *in vitro* susceptibility experiments and the improved fitness of the Δ*osmB* strain in the mouse model, independent of drug treatment (Fig 4B, 4C and 4E). These data may suggest there is redundancy in lipoprotein expression, and that expression of other lipoproteins are sufficient to compensate for a loss of *osmB*. Consistent with this, *osmB* was not required high osmolarity conditions in either stationary or exponential phase (S3 Fig). *osmB* expression increases during a transition to stationary phase growth, and in response to changes in osmolarity, which would presumably promote increased membrane fluidity under these conditions [44,45,55]. Multiple *osm* genes are expressed under these conditions, and subsets of these could potentially compensate for loss of *osmB* [42]. OsmB also has a role in repairing the *E*. *coli* outer membrane after stress, which would suggest that OsmB could play some role in membrane repair after damage caused by antibiotic treatment [44,55,57]. Although our data suggest that OsmB does not significantly contribute to Dox susceptibility or tolerance in the conditions tested, and loss of *osmB* only had a slight impact on the accumulation of Dox within bacterial cells, OsmB may still play some other role that helps the cell persist during antibiotic exposure. Conversely, because OsmB has a predicted role in membrane permeability, it is also possible that the increased growth of Δ*osmB* is due to heightened nutrient uptake due to altered membrane dynamics. This was part of the rationale for including *in vitro* competitive growth experiments in this manuscript alongside single strain growth; if any mutant strains have altered nutrient uptake, then they may grow differently alone, compared to the presence of the WT strain. This would have implications for growth of the two strains together in the mouse model, which is also why we assessed competitive growth *in vitro*.

*ompF* porin expression was heightened during Dox exposure, which impacted antibiotic accumulation within the bacterial cell, and indicated that OmpF may promote diffusion of doxycycline out of the cell (Fig 5B and 5G). These results were surprising; we had expected to see decreased expression of porins during Dox exposure, since OmpF has been shown to promote passive uptake of multiple different antibiotics through the outer membrane, including tetracyclines [24,36,38,41]. Increased OmpF expression would have been expected to increase the amount of Dox influx into the cell, and negatively impact bacterial survival. The accumulation of Dox in the *ompF* deletion mutant instead indicates this porin may also have a role in passive diffusion of tetracyclines back out of the bacterial cell. Notably, the phenotypes with Δ*ompF* were most pronounced at low levels of Dox, where you might expect more of a role of passive diffusion in lowering intracellular antibiotic levels [36,38]. Expression of efflux pumps promotes tetracycline resistance by pumping antibiotics out of the bacterial cell [15,24,36], and we had also expected to see heightened expression of efflux pumps in response to Dox exposure. It is possible that we might see expression of efflux pumps in response to heightened levels of Dox, and it is likely that many additional pathways are differentially regulated in response to more stressful, inhibitory concentrations of Dox. It will be interesting to further characterize the transcriptional changes that occur during exposure to heightened doses of Dox, and also probe more deeply the bacterial responses to Dox treatment within host tissues.

Another surprising result was the comparison in phenotypes of the Δ*ompF* and Δ*osmB* Δ*ompF* strains. After seeing subtle phenotypes with the Δ*ompF* strain, we had expected to see similar or stronger evidence of antibiotic susceptibility with the Δ*osmB* Δ*ompF* strain. Instead, the deletion of *osmB* appeared to partially rescue the Δ*ompF* phenotype, possibly indicating aberrant *osmB* expression, detrimental mislocalization of OsmB, or misregulation of other outer membrane components, occurs in the absence of OmpF [58]. A direct interaction between OmpF and OsmB has not been shown, however it is possible OsmB could function like other lipoproteins, and contribute to phospholipid transport through interactions with porins [58]. In which case, loss of OmpF could have additional effects on the outer membrane, which could explain a stronger phenotype with the Δ*ompF* single deletion strain. Some of the additional impacts of OmpF function on the outer membrane may have impacted our ability to rescue *ompF* directly. The stress of losing these additional functions of OmpF, in addition to loss of OsmB, may have also resulted in additional genetic changes in the Δ*osmB* Δ*ompF* background, impacting the phenotypes of rescued strains derived from this parent strain (S4 Fig). It is also interesting to think about the expression of these two genes, and what it means to see both genes upregulated in response to Dox. Both *osmB* and *ompF* are regulated by the alternative sigma factor, RpoS, which promotes expression of genes in stationary phase [41,55]. However, *osmB* expression occurs independently of EnvZ and OmpR, which are required for *ompF* expression, again suggesting *osmB* and *ompF* are regulated independently [42]. It has also been shown that *osmB* is expressed under high osmolarity, conditions that repress *ompF* expression, providing further evidence that expression of these genes does not typically occur under the same conditions [42]. These data indicate that Dox exposure does not mimic different changes in osmolarity, and while it is possible that RpoS-dependent gene expression could occur in response to this stress, we did not see heightened expression of the RpoS-regulated gene, *dps* (S1 Fig).

Overexpression of *tusB* resulted in a significant impact on bacterial viability (Figs 6I–6K and 7C), suggesting that tRNA modification by *tusB*, and resulting impacts on translational machinery, may play an important role in promoting tolerance. We believe this may be the first time bactericidal activity of Dox has been described, however it is possible that other genetic modifications could also promote bactericidal activity of this typically bacteriostatic antibiotic [14,16]. Tetracyclines bind reversibly to the 16S rRNA component of the ribosomal small subunit, inhibiting translation [25]. TusB function, in contrast, promotes the stabilization of the wobble position of specific tRNAs for proper incorporation of glutamine (Gln), glutamate (Glu), and lysine (Lys) residues into growing polypeptide chains [33]. Because of the role of TusB relatively early in this 2-thiouridylation cascade, it is unlikely that TusB remains associated with tRNAs as they enter ribosomes, and so Dox and TusB should not both be interacting with ribosomes directly [33,34]. Previous studies have shown that bacteriostatic antibiotics can have bactericidal activity if they bind their target with higher affinity [14,16], however we believe that ribosomal inhibition by high levels of tRNA modifications may underly our observed phenotype. We believe that the toxicity associated with heightened TusB expression during Dox exposure could be linked to heightened activity of the cascade, increased levels of 2-thiouridylation, and potential stalling at Gln, Glu, and Lys codons for proper incorporation of these amino acids. We hypothesize that heightened *tusB* expression results in ribosomal stalling, which synergizes with ribosomal inhibition by Dox to more effectively shutdown translational machinery, resulting in bacterial lethality at concentrations of Dox that are typically bacteriostatic. Conversely, in WT cells that downregulate *tusB* expression, we would expect cells to translate proteins more quickly, albeit with errors in incorporation of Gln, Glu, and Lys residues. We are very interested in pursuing the mechanism underlying the TusB phenotype in future studies, to better understand how modulation of translation may promote increased Dox toxicity, and ultimately improve efficacy of Dox treatment.

It is also important to note there was a slight fitness cost associated with the *P*_*tetA*_::*tusB* construct *in vivo* that may be due to increased *tusB* expression within mouse tissues prior to Dox administration. Our qRTPCR data was consistent with this, showing this strain has increased levels of *tusB* expression prior to Dox treatment (Fig 6B). This impact on fitness was specific to the host environment, since the *P*_*tetA*_::*tusB* strain replicated as well as the WT strain in culture (Fig 6G and 6H). For future studies we plan to generate a panel of different *tusB* strains to examine the impact of constitutive *tusB* expression, induction from a single copy *P*_*tetA*_::*tusB* construct, and deletion of *tusB* on Dox susceptibility.

We have found in previous studies that bacteria within microcolonies experience different microenvironments, resulting in differing levels of stress responses [52,54]. We have also shown that Dox does not diffuse evenly across microcolonies at this 40mg/kg single injection dosage [18], which suggests that bacteria may also respond to Dox in a heterogeneous manner within host tissues. It remains unclear if the genes we have studied here are differentially regulated within the same cells, or if distinct subpopulations differentially express these gene products. In future experiments, we plan to explore this idea using fluorescent reporters to determine whether individual cells differentially regulate genes of interest in response to Dox, and also develop single cell assays to better understand and characterize the extent of heterogeneity in the response to antibiotics. It will also be important to characterize the response to Dox specifically within host tissues. For additional future studies, we plan to isolate surviving bacterial cells from the host environment to determine which pathways promote persistence in the host, and compare these to the specific responses to Dox in culture.

## Materials and methods

### Ethics statement

The animal experiments within this manuscript were approved by the Johns Hopkins Institutional Animal Care and Use Committee under protocol number: MO19H418. Experiments with *Y*. *pseudotuberculosis* were approved by the Johns Hopkins Institutional Biosafety Committee under protocol numbers P1611010206 and DN1611010106.

### Bacterial strains & growth conditions

The WT *Y*. *pseudotuberculosis* strain, IP2666, was used throughout this study. For all *in vitro* experiments, overnight cultures of bacteria were grown for 16–18 h in lysogeny broth (LB) at 26°C with rotation. Bacteria were then sub-cultured 1:100 into fresh LB and incubated at 37°C with rotation for experiments. Overnight cultures were considered stationary phase, exponential phase cells were prepared as described below. Doxycycline was added to the samples at the indicated concentrations. For *in vivo* experiments, bacteria were cultured overnight as described above, in 2xYT broth (LB with 2x yeast extract and tryptone) [18,52]. Bacteria were then diluted in sterile phosphate-buffered saline solution (PBS) to a final concentration of 10^3^ CFU/100μl.

### qRT-PCR to detect bacterial transcripts in broth-grown cultures

Bacterial cells were grown in the presence or absence of antibiotics for the indicated time points, pelleted, resuspended in Buffer RLT (QIAGEN) + ß-mercaptoethanol, and RNA was isolated using the RNeasy kit (QIAGEN). DNA contamination was eliminated using either the Turbo DNA-free kit (Invitrogen) or the on-column RNase-free DNase set (QIAGEN). RNA was reverse transcribed using M-MLV reverse transcriptase and random primers (Invitrogen), in the presence of the RNase inhibitor, RnaseOUT (Invitrogen). Approximately 30 ng cDNA was used as a template in qPCR reactions with 0.5 μM of forward and reverse primers [52] and Power SYBR Green PCR Master mix (Applied Biosystems). Control samples were prepared that lacked reverse transcriptase to confirm genomic DNA was eliminated from samples. qPCR reactions were carried out using the StepOnePlus Real-Time PCR system, and comparisons were obtained using the ΔΔC_T_ and 2^-ΔCt^ method (Applied Biosystems) for relative expression. Kits were used according to manufacturers’ protocols.

### RNA-seq

Overnight cultures of WT *Y*. *pseudotuberculosis* were diluted 1:100 into fresh media and grown at 37°C in the presence or absence of 0.1μg/ml Dox. At 2h and 4h post-treatment, approximately 10^5^ bacterial cells were collected from cultures, and RNA was isolated as described above. RNA was prepared for RNA-seq analysis using a RNAtag-seq approach [35]. Following sequencing, reads were aligned using the *Y*. *pseudotuberculosis* strain YPIII fully annotated genome (genome: NC_010465, virulence plasmid: NZ_LT596222). Relative abundance changes in transcript levels were assessed using a DESeq2 method of pairwise comparisons between treatment groups [59]. An adjusted p-value of 0.05 was considered significant, and we focused on genes differentially regulated ≥2 fold for validation experiments and downstream assays.

### Murine model of systemic infection

Jackson Laboratories (Bar Harbor, ME) provided 6–8 week-old female C57BL/6 mice and the animal experiments were approved by the Johns Hopkins University Institutional Animal Care and Use Committee (Protocol #: MO19H418). Mice were inoculated intravenously via tail vein injection with 10^3^ CFU *Y*. *pseudotuberculosis* in 100μl for all experiments [52]. At 48 hours post-inoculation, mice were treated with 40 mg/kg doxycycline (720μg in 100μl sterile PBS) via intraperitoneal injection [18]. Spleens were harvested at the indicated timepoints post-treatment and either homogenized to quantify CFUs, stabilized in RNALater for RNA isolation, or fixed in 4% paraformaldehyde (PFA) for histology. Tissue was fixed by incubating 24h in 4% PFA at 4° C. Animals were euthanized by lethal isoflurane asphyxiation followed by cervical dislocation and vital organ removal.

### qRT-PCR to detect bacterial transcripts from infected tissues

At the indicated timepoints, spleens were harvested and immediately submerged in RNALater to stabilize transcripts, incubated at 4°C three weeks, then tissue was stored at –80°C prior to RNA isolation. Tissue was then thawed, added to Buffer RLT (QIAGEN) + ß-mercaptoethanol (200μl/10mg tissue), and homogenized. Cell debris was pelleted, and lysate was used for RNA isolation as described above. DNA contamination was eliminated using the Turbo DNA-free kit (Invitrogen). The MicrobEnrich kit (Invitrogen) was used to deplete mouse mRNAs, tRNAs and rRNAs. Approximately 500ng RNA was used in reverse transcription reactions, and approximately 25ng cDNA were used in qPCR reactions, as described above.

### Generation of deletion and rescued strains

*osmB* and *ompF* deletion strains were generated by amplifying the start codon + 3 downstream codons, the 3’ terminal 3 codons + the stop codon and fusing these fragments to generate a start + 6 aa + stop deletion construct for each gene. Deletion constructs were amplified with 500 base pairs flanking sequence on each side, cloned into the suicide vector, pSR47S, and conjugated into *Y*. *pseudotuberculosis* [60]. Sucrose selection was used to select for bacteria that had incorporated the desired mutation after a second recombination event [60]. PCR, sequencing, and qRT-PCR were used to confirm deletion strains. To mark mutant cells with fluorescence, we also generated deletion strains that contained the previously described, *yopE*::*mCherry* reporter [52,53]. Rescued strains were generated by amplifying the gene of interest with 500 base pairs flanking sequence on each side, inserting this into pSR47S, conjugating into *Y*. *pseudotuberculosis* deletion strains, and selecting bacteria that had integrated the WT copy of the gene into the chromosome. Rescue was confirmed by PCR and sequencing.

### Generation of the TetON reporter strains and P_tetA_::tusB construct

The TetON reporter (*P*_*tetA*_::*mCherry-ssrA*) was previously described [18], and was constructed by fusing *tetR* through the *P*_*tetA*_ promoter to a ssrA-tagged destabilized variant of *mCherry* [61,62]. The TetON reporter was expressed from the low copy pMMB67EH plasmid and transformed into WT and mutant strains by electroporation [60]. The constitutive GFP plasmid used alongside TetON in mouse infections has been previously described [18,52,53]. This GFP plasmid was also used to mark the WT strain for *in vitro* and *in vivo* competition experiments with the *osmB* and *ompF* deletion strains. *P*_*tetA*_::*tusB* was generated the same way as the TetON reporter; the *tetR* through *P*_*tetA*_ fragment was fused to *tusB*. *P*_*tetA*_::*tusB* was inserted into the low copy pACYC184 plasmid and electroporated into WT *Y*. *pseudotuberculosis*, as previously described [60]. To mark the *P*_*tetA*_::*tusB* strain with fluorescence, we also transformed the *P*_*tetA*_::*tusB* plasmid into a WT *yopE*::*mCherry* reporter strain [52,53].

### Antibiotic susceptibility assays

Antibiotic susceptibility of mutant strains was assessed using three *in vitro* approaches: growth inhibition, viability, and competitive survival. Growth inhibition was assessed by diluting overnight cultures (1:100) into fresh LB containing increasing doses of Dox (0.01–1μg/ml), and quantifying absorbance (600nm) every 2h using a microplate reader. Cultures were incubated for 8h at 37° C with rotation. Viability was assessed with exponential phase cells, which were subcultured from overnight cultures (1:100) into fresh LB and rotated at 26° C for 2h. Exponential phase cells were then transferred to fresh LB containing increasing doses of Dox (0.1–4μg/ml), and incubated 4h at 37° C with rotation. Cells were plated for viability at the time of Dox addition (0h), 2h, and 4h post-treatment. Competitive survival was assessed with exponential phase cells prepared as described above, and cells were co-cultured in wells of 96 well plates with increasing doses of Dox. Plates were incubated at 37° C on an orbital shaker (VWR). Single strain controls were grown in parallel to ensure growth and Dox sensitivity was similar in 96 well plates compared to test tubes. Cells were plated to quantify CFUs of each strain at the time of Dox addition (0h), 2h, and 4h post-treatment. Chloramphenicol (Cm) resistance was used to quantify the number of WT cells relative to total bacterial numbers for the experiments with deletion strains. For the WT vs. *P*_*tetA*_::*tusB* experiments, the *tusB* strain was marked with Cm resistance.

### High osmolarity experiments

Overnight cultures were considered stationary phase, and addition of 1M sorbitol, 1M NaCl, or vehicle control (equivalent volume dH_2_O) were added directly to cultures. For experiments with exponential phase cells, aliquots of overnight cultures were washed in LB without NaCl (yeast extract and tryptone only) and subcultured (1:100 dilution) into LB without NaCl. Cultures were incubated 2h at 26° C with rotation before addition of the indicated amounts of NaCl (0.5M, 1M) or vehicle control (equivalent volume dH_2_O). Absorbance (600nm) was used to detect changes in the density of cultures, values were normalized to the starting absorbance of each culture.

### NO donor experiments

Experiments with the NO donor, DETA-NONOate (Cayman Chemical), were performed with exponential phase cells, prepared as described above. Exponential phase cells were transferred to fresh LB in the presence or absence of 2.5mM DETA-NONOate in 96 well plate. Cultures were immediately plated to determine the starting CFU, then incubated 4h at 37° C with orbital shaking, and plated to determine the final CFU. Fold change in CFU was calculated for each culture.

### Fluorescence microscopy—Host tissues

After fixation with PFA, spleens were frozen-embedded in optimal cutting temperature (O.C.T.) compound (Tissue-Tek, VWR). 10 μm thick sections were obtained with a cryostat microtome (Microm HM 505e) and transferred to charged microscope slides (VWR). Slides were stored at –80° C, then thawed in PBS at room temperature, washed 3x in PBS, and stained with Hoechst (1:10,000 dilution in PBS) for 5 minutes. Slides were then washed 3x with PBS and coverslips were mounted with ProLong Gold (Life Technologies). One section was imaged for each tissue. Tissues were imaged with a Zeiss Axio Observer 7 (Zeiss) inverted fluorescent microscope with an Apotome.2 (Zeiss) with the 63x oil immersion objective. Images of the sections were captured with an Axiocam 702 mono camera (Zeiss).

### Antibiotic permeability assays

Exponential phase cultures of TetON-containing strains (WT, Δ*osmB*, Δ*ompF*, Δ*osmB ΔompF*) were incubated in the absence or presence of increasing doses of Dox (0.01–1μg/ml) for 4h at 37° C with rotation. Approximately 10^6^ cells were pelleted and fixed in 4% PFA at 4° C for 18h (overnight). Fixed cells were pelleted, PFA was removed, and cells were resuspended in 20μl sterile PBS. 5μl of each sample was imaged on 1% agarose (in PBS) gel pads. 5 fields of view were captured for each sample, and single cell fluorescence was quantified using Volocity software, as described below. This resulted in approximately 500–800 total cells imaged across three biological replicates, or approximately 150–300 cells per condition, per replicate.

### Image analysis

Volocity image analysis software was used to quantify microcolony areas as previously described [18,52]. Briefly, either GFP or mCherry signal were used to detect individual bacteria and quantify total area of each microcolony. One cross-section/tissue was imaged to represent each spleen. Dox permeability was detected using TetON reporter expression, which was quantified using Volocity to measure single cell mCherry fluorescence. For *in vitro* experiments, relative mCherry signal was calculated by dividing the mCherry fluorescence value of each cell by the average mCherry value of untreated WT cells, which were used to set a baseline fluorescence value. For *in vivo* experiments, relative Dox accumulation was quantified by dividing the mean mCherry signal across a microcolony, by the mean signal of constitutive GFP, as previously described [18].

## Supporting information

S1 Fig Known stress response genes are not differentially regulated in response to doxycycline. Cultures of WT *Y*. *pseudotuberculosis* were incubated in the presence or absence of 0.1μg/ml Dox, RNA was isolated and transcripts were detected by qRT-PCR. Log2 fold change values were calculated relative to untreated cells, horizontal dotted lines depict average values for untreated cells (0log2), and 2-fold changes. Data represents four biological replicates.
(TIF)

S2 Fig *ompF* rescued strains partially rescue phenotypes. Exponential phase cultures of rescued strains (Δ*osmB ompF*^*+*^, *osmB*^*+*^ Δ*ompF*, *osmB*^*+*^ *ompF*^*+*^) were treated with the indicated concentrations of Dox to assess **(A)** growth inhibition and **(B)** competitive survival. **(A)** Strains were incubated with 0.01μg/ml Dox and growth inhibition was assessed based on absorbance (A_600nm_) at the indicated timepoints (time: hours, h). Data represents the mean and standard deviation of four biological replicates. **(B)** Competitive survival in the presence of the indicated concentrations of Dox; rescued strains were tested alongside the WT GFP^+^ strain. Competitive index (CI): CFUs of the rescue/WT divided by the ratio of rescue/WT in the culture at time 0h. Values above 1 indicate the rescue preferentially survives, values less than 1 indicate the WT preferentially survives. Dotted line: value of 1, equal fitness. Dots: biological replicates, lines connect biological replicates sampled across the timepoints, three biological replicates shown. Statistics: Two-way ANOVA with Tukey’s multiple comparison test, **(A)**: comparisons made relative to the *osmB*^*+*^ *ompF*^*+*^ strain; **(B)**: comparisons made between 4h treatment CI values. n.s.: not significant.
(TIF)

S3 Fig The Δ*osmB* strain does not have increased sensitivity to high osmolarity culture conditions. **(A)** Stationary and **(B)** exponential phase cultures of the WT, Δ*osmB*, and the *osmB*^*+*^ (*osmB* rescue) strains were incubated under the indicated high osmolarity conditions (1M sorbitol, 0.5M and 1M NaCl). Sensitivity was assessed based on absorbance (A_600nm_) at the indicated timepoints (time: hours, h). Data was normalized to the starting absorbance value of each culture (value of 1, dotted line), and is shown as log_10_ fold change. Controls depict vehicle controls. Data represents the mean and standard deviation of two biological replicates. Statistics: Two-way ANOVA (no statistical significance), post-tests not performed.
(TIF)

S4 Fig Rescued mutant strains retain altered antibiotic accumulation. Δ*osmB* Δ*ompF*, Δ*osmB ompF*^*+*^, *osmB*^*+*^ Δ*ompF*, *osmB*^*+*^ *ompF*^*+*^, and WT strains transformed with the TetON reporter plasmid were incubated with the indicated concentrations of Dox for 4h. mCherry fluorescence was detected within individual cells by fluorescence microscopy. Relative mCherry was calculated by normalizing to a WT untreated average cell value (imaged in parallel). Data represents the mean and standard deviation of three biological replicates. Statistics: Kruskal-Wallis one-way ANOVA, Dunn’s post-test. ****p < .0001, ***p < .001, *p < .05, n.s.: not significant.
(TIF)

## References

[1] Bigger J. Treatment of Staphylococcal infections with penicillin by intermittent sterilisation. Lancet. 1944;244(6320):497–500.

[2] McCune R Jr, Tompsett R. Fate of *Mycobacterium tuberculosis* in mouse tissues as determined by the microbial enumeration technique. I. The persistence of drug-susceptible tubercle bacilli in the tissues despite prolonged antimicrobial therapy. J Exp Med. 1956;104(5):737–62. doi: 10.1084/jem.104.5.737 13367341 PMC2136613

[3] Balaban N, Helaine S, Lewis K, Ackermann M, Aldridge B, Andersson D, et al. Definitions and guidelines for research on antibiotic persistence. Nat Rev Microbiol. 2019;17(77):441–8. 30980069 10.1038/s41579-019-0196-3 PMC7136161

[4] Brauner A, Fridman O, Gefen O, Balaban N. Distinguishing between resistance, tolerance and persistence to antibiotic treatment. Nat Rev Microbiol. 2016;14(5):320–30. doi: 10.1038/nrmicro.2016.34 27080241

[5] Brauner A, Shoresh N, Fridman O, Balaban N. An Experimental Framework for Quantifying Bacterial Tolerance. Biophys J. 2017;112(12):2664–71. doi: 10.1016/j.bpj.2017.05.014 28636922 PMC5479142

[6] Liu J, Gefen O, Ronin I, Bar-Meir M, Balaban N. Effect of tolerance on the evolution of antibiotic resistance under drug combinations. Science. 2020;367(6474):200–4. doi: 10.1126/science.aay3041 31919223

[7] Gollan B, Grabe G, Michaux C, Helaine S. Bacterial Persisters and Infection: Past, Present, and Progressing. Annu Rev Microbiol. 2019;73:359–85. doi: 10.1146/annurev-micro-020518-115650 31500532

[8] Dörr T, Vulic M, Lewis K. Ciprofloxacin causes persister formation by inducing the TisB toxin in *Escherichia coli*. PLoS Biol. 2010;8. doi: 10.1371/journal.pbio.1000317 20186264 PMC2826370

[9] Johnson P, Levin B. Pharmacodynamics, population dynamics, and the evolution of persistence in *Staphylococcus aureus*. PLoS Genet. 2013;9(1). 10.1371/journal.pgen.1003123 PMC3536638 23300474

[10] Dörr T, Davis B, Waldor M. Endopeptidase-mediated beta lactam tolerance. PLoS Pathog. 2015;11(4). doi: 10.1371/journal.ppat.1004850 25884840 PMC4401780

[11] Handwerger S, Tomasz A. Antibiotic tolerance among clinical isolates of bacteria. Rev Infect Dis. 1985;7(3):368–86. doi: 10.1093/clinids/7.3.368 3895353

[12] Zalis E, Nuxoll A, Manuse S, Clair G, Radlinski L, Conlon B, et al. Stochastic variation in expression of the tricarboxylic acid cycle produces persister cells. MBio. 2019;10(5):e01930–19. doi: 10.1128/mBio.01930-19 31530676 PMC6751062

[13] Lewis K. Persister cells, dormancy and infectious disease. Nat Rev Microbiol. 2007;5(1):48–56. doi: 10.1038/nrmicro1557 17143318

[14] Levin B, McCall I, Perrot V, Weiss H, Ovesepian A, Baquero F. A Numbers Game: Ribosome Densities, Bacterial Growth, and Antibiotic-Mediated Stasis and Death. mBio. 2017;8(1):e02253–16. doi: 10.1128/mBio.02253-16 28174311 PMC5296603

[15] Schnappinger D, Hillen W. Tetracyclines: antibiotic action, uptake, and resistance mechanisms. Arch Microbiol. 1996;165(6):359–69. doi: 10.1007/s002030050339 8661929

[16] Svetlov M, Vázquez-Laslop N, Mankin A. Kinetics of drug-ribosome interactions defines the cidality of macrolide antibiotics. Proc Natl Acad Sci. 2017;114(52):13673–8. doi: 10.1073/pnas.1717168115 29229833 PMC5748224

[17] Baquero F, Levin B. Proximate and ultimate causes of the bactericidal action of antibiotics. Nat Rev Microbiol. 2021;19(2):123–32. doi: 10.1038/s41579-020-00443-1 33024310 PMC7537969

[18] Raneses J, Ellison A, Liu B, Davis K. Subpopulations of stressed *Y*. *pseudotuberculosis* preferentially survive doxycycline treatment within host tissues. mBio. 2020;11(4):e00901–20. doi: 10.1128/mBio.00901-20 32753491 PMC7407081

[19] Mwengee W, Butler T, Mgema S, Mhina G, Almasi Y, Bradley C, et al. Treatment of plague with gentamicin or doxycycline in a randomized clinical trial in Tanzania. Clin Infect Dis. 2006;42(5):614–21. doi: 10.1086/500137 16447105

[20] Bonacorsi S, Scavizzi M, Guiyoule A, Amouroux J, Carniel E. Assessment of a fluoroquinolone, three beta-lactams, two aminoglycosides, and a cycline in treatment of murine *Yersinia pestis* infection. Antimicrob Agents Chemother. 1994;38(3):481–6. doi: 10.1128/AAC.38.3.481 8203841 PMC284484

[21] Hoogkamp-Korstanje J. Antibiotics in *Yersinia enterocolitica* infections. J Antimicrob Chemother. 1987;20(1):123–31. doi: 10.1093/jac/20.1.123 3497913

[22] Lemaitre B, Mazigh D, Scavizzi M. Failure of beta-lactam antibiotics and marked efficacy of fluoroquinolones in treatment of murine *Yersinia pseudotuberculosis* infection. Antimicrob Agents Chemother. 1991;35(9):1785–90. doi: 10.1128/AAC.35.9.1785 1952849 PMC245269

[23] Stolk-Engelaar V, Meis J, Mulder J, Loeffen F, Hoogkamp-Korstanje J. *In vitro* antimicrobial susceptibility of *Yersinia enterocolitica* isolates from stools of patients in The Netherlands from 1982–1991. J Antimicrob Chemother. 1995;36(5):839–43. doi: 10.1093/jac/36.5.839 8626266

[24] Chopra I, Roberts M. Tetracycline antibiotics: mode of action, applications, molecular biology, and epidemiology of bacterial resistance. Microbiol Mol Biol Rev. 2001;65(2):232–60. doi: 10.1128/MMBR.65.2.232-260.2001 11381101 PMC99026

[25] Chukwudi C. rRNA Binding Sites and the Molecular Mechanism of Action of the Tetracyclines. Antimicrob Agents Chemother. 2016;60(8):4433–41. doi: 10.1128/AAC.00594-16 27246781 PMC4958212

[26] Moreno-Gamez S, Hill A, Rosenbloom D, Petrov D, Nowak M, Pennings P. Imperfect drug penetration leads to spatial monotherapy and rapid evolution of multidrug resistance. Proc Natl Acad Sci. 2015;112(22):E2874–83. doi: 10.1073/pnas.1424184112 26038564 PMC4460514

[27] Prideaux B, Via L, Zimmerman M, Eum S, Sarathy J, O’Brien P, et al. The association between sterilizing activity and drug distribution into tuberculosis lesions. Nat Med. 2015;21(10):1223–7. doi: 10.1038/nm.3937 26343800 PMC4598290

[28] Heine W, Beckstette M, Heroven A, Thiemann S, Heise U, Nuss A, et al. Loss of CNFY toxin-induced inflammation drives *Yersinia pseudotuberculosis* into persistency. PLoS Pathog. 2018;14(2):e1006858. doi: 10.1371/journal.ppat.1006858 29390040 PMC5811047

[29] Capron J, Delamarre J, Delcenserie R, Gineston J, Dupas J, Lorriaux A. Liver absess complicating *Yersinia pseudotuberculosis* ileitis. Gastroenterology. 1981;81(1):150–2. 7016657

[30] Paff J, Triplett D, Saari T. Clinical and laboratory aspects of *Yersinia pseudotuberculosis* infections, with a report of two cases. Am J Clin Pathol. 1976;66(1):101–10. doi: 10.1093/ajcp/66.1.101 779444

[31] Russell P, Eley S, Green M, Stagg A, Taylor R, Nelson M, et al. Efficacy of doxycycline and ciprofloxacin against experimental *Yersinia pestis* infection. J Antimicrob Chemother. 1998;41(2):301–5. doi: 10.1093/jac/41.2.301 9533478

[32] Balaban N, Merrin J, Chait R, Kowalik L, Leibler S. Bacterial persistence as a phenotypic switch. Science. 2004;305(5690):1622–5. doi: 10.1126/science.1099390 15308767

[33] Ikeuchi Y, Shigi N, Kato J, Nishimura A, Suzuki T. Mechanistic insights into sulfur relay by multiple sulfur mediators involved in thiouridine biosynthesis at tRNA wobble positions. Mol Cell. 2006;21(1):97–108. doi: 10.1016/j.molcel.2005.11.001 16387657

[34] Numata T, Fukai S, Ikeuchi Y, Suzuki T, Nureki O. Structural basis for sulfur relay to RNA mediated by heterohexameric TusBCD complex. Structure. 2006;14(2):357–66. doi: 10.1016/j.str.2005.11.009 16472754

[35] Shishkin A, Giannoukos G, Kucukural A, Ciulla D, Busby M, Surka C, et al. Simultaneous generation of many RNA-seq libraries in a single reaction. Nat Methods. 2015;12(4):323–5. doi: 10.1038/nmeth.3313 25730492 PMC4712044

[36] Masi M, Réfregiers M, Pos K, Pagés J. Mechanisms of envelope permeability and antibiotic influx and efflux in Gram-negative bacteria. Nat Microbiol. 2017;2. doi: 10.1038/nmicrobiol.2017.1 28224989

[37] Sánchez-Romero M, Casadesús J. Contribution of phenotypic heterogeneity to adaptive antibiotic resistance. Proc Natl Acad Sci. 2014;111(1):355–60. doi: 10.1073/pnas.1316084111 24351930 PMC3890857

[38] Vergalli J, Bodrenko I, Masi M, Moynié L, Acosta-Gutiérrez S, Naismith J, et al. Porins and small-molecule translocation across the outer membrane of Gram-negative bacteria. Nat Rev Microbiol. 2020;18(3):164–76. doi: 10.1038/s41579-019-0294-2 31792365

[39] Lucchetti J, Fracasso C, Balducci C, Passoni A, Forloni G, Salmona M, et al. Plasma and brain concentrations of doxycycline after single and repeated doses in wild-type and APP23 mice. J Pharmacol Exp Ther. 2019;368(1):32–40. doi: 10.1124/jpet.118.252064 30396916

[40] Hancock R, Woodruff W. Roles of porin and beta-lactamase in beta-lactam resistance of *Pseudomonas aeruginosa*. Rev Infect Dis. 1988;10(4):770–5. doi: 10.1093/clinids/10.4.770 2460909

[41] Pagés J, James C, Winterhalter M. The porin and the permeating antibiotic: a selective diffusion barrier in Gram-negative bacteria. Nat Rev Microbiol. 2008;6(12):893–903. doi: 10.1038/nrmicro1994 18997824

[42] Gutierrez C, Barondess J, Manoil C, Beckwith J. The use of transposon TnphoA to detect genes for cell envelope proteins subject to a common regulatory stimulus. Analysis of osmotically regulated genes in *Escherichia coli*. J Mol Biol. 1987;195(2):289–97. doi: 10.1016/0022-2836(87)90650-4 2821274

[43] Jung J, Gutierrez C, Villarejo M. Sequence of an osmotically-inducible lipoprotein gene. J Bacteriol. 1989;171(1):511–20. doi: 10.1128/jb.171.1.511-520.1989 2644204 PMC209616

[44] Boulanger A, Francez-Charlot A, Conter A, Castanié-Cornet M, Cam K, Gutierrez C. Multistress regulation in *Escherichia coli*: expression of *osmB* involves two independent promoters responding either to sigmaS or to the RcsCDB His-Asp phosphorelay. J Bacteriol. 2005;187(9):3282–6. doi: 10.1128/JB.187.9.3282-3286.2005 15838058 PMC1082829

[45] Jung J, Gutierrez C, Martin F, Ardourel M, Villarejo M. Transcription of *osmB*, a gene encoding an *Escherichia coli* lipoprotein, is regulated by dual signals. Osmotic stress and stationary phase. J Biol Chem. 1990;265(18):10574–81. 1693921

[46] Fridman O, Goldberg A, Ronin I, Shoresh N, Balaban N. Optimization of lag time underlies antibiotic tolerance in evolved bacterial populations. Nature. 2014;513(7518):418–21. doi: 10.1038/nature13469 25043002

[47] Mueller M, de la Peña A, Derendorf H. Issues in pharmacokinetics and pharmacodynamics of anti-infective agents: kill curves versus MIC. Antimicrob Agents Chemother. 2004;48(2):369–77. doi: 10.1128/AAC.48.2.369-377.2004 14742182 PMC321563

[48] Wiegand I, Hilpert K, Hancock R. Agar and broth dilution methods to determine the minimal inhibitory concentration (MIC) of antimicrobial substances. Nat Protoc. 2008;3(2):163–75. doi: 10.1038/nprot.2007.521 18274517

[49] Russell P, Eley S, Bell D, Manchee R, Titball R. Doxycycline or ciprofloxacin prophylaxis and therapy against experimental *Yersinia pestis* infection in mice. J Antimicrob Chemother. 1996;37(4):769–74. doi: 10.1093/jac/37.4.769 8722542

[50] Heine H, Bassett J, Miller L, Hartings J, Ivins B, Pitt M, et al. Determination of antibiotic efficacy against *Bacillus anthracis* in a mouse aerosol challenge model. Antimicrob Agents Chemother. 2007;51(4):1373–9. doi: 10.1128/AAC.01050-06 17296745 PMC1855446

[51] Davis K, Krupp J, Clark S, Isberg R. Iron-sulfur cluster repair contributes to *Yersinia pseudotuberculosis* survival within deep tissues. Infect Immun. 2019;87(10). doi: 10.1128/IAI.00533-19 31331956 PMC6759291

[52] Davis K, Mohammadi S, Isberg R. Community behavior and spatial regulation within a bacterial microcolony in deep tissue sites serves to protect against host attack. Cell Host Microbe. 2015;17(1):21–31. doi: 10.1016/j.chom.2014.11.008 25500192 PMC4669952

[53] Crimmins G, Mohammadi S, Green E, Bergman M, Isberg R, Mecsas J. Identification of MrtAB, an ABC transporter specifically required for *Yersinia pseudotuberculosis* to colonize the mesenteric lymph nodes. PLoS Pathog. 2012;8(8):e1002828. doi: 10.1371/journal.ppat.1002828 22876175 PMC3410872

[54] Patel P, O’Hara B, Aunins E, Davis K. Modifying TIMER to generate a slow-folding DsRed derivative for optimal use in quickly-dividing bacteria. PLoS Pathog. 2021;17(7). doi: 10.1371/journal.ppat.1009284 34214139 PMC8291646

[55] Charoenwong D, Andrews S, Mackey B. Role of *rpoS* in the development of cell envelope resilience and pressure resistance in stationary-phase *Escherichia coli*. Appl Environ Microbiol. 2011;77(15):5220–9. doi: 10.1128/AEM.00648-11 21705547 PMC3147466

[56] Schlünzen F, Zarivach R, Harms J, Bashan A, Tocilj A, Albrecht R, et al. Structural basis for the interaction of antibiotics with the peptidyl transferase centre in eubacteria. Nature. 2001;4133(6858):814–21. doi: 10.1038/35101544 11677599

[57] Guest R, Raivio T. Role of the Gram-Negative Envelope Stress Response in the Presence of Antimicrobial Agents. Trends Microbiol. 2016;24(5):377–90. doi: 10.1016/j.tim.2016.03.001 27068053

[58] Abellón-Ruiz J, Kaptan S, Baslé A, Claudi B, Bumann D, Kleinekathöfer U, et al. Structural basis for maintenance of bacterial outer membrane lipid asymmetry. Nat Microbiol. 2017;2(12):1616–23. doi: 10.1038/s41564-017-0046-x 29038444

[59] Love M, Huber W, Anders S. Moderated estimation of fold change and dispersion for RNA-seq data with DESeq2. Genome Biol. 2014;15(12). doi: 10.1186/s13059-014-0550-8 25516281 PMC4302049

[60] Davidson R, Davis K. *Yersinia pseudotuberculosis*: Cultivation, Storage, and Methods for Introducing DNA. Curr Protoc Microbiol. 2020;59(1). 10.1002/cpmc.122 33079471

[61] Andersen J, Sternberg C, Poulsen L, Bjorn S, Givskov M, Molin S. New unstable variants of green fluorescent protein for studies of transient gene expression in bacteria. Appl Environ Microbiol. 1998;64(6):2240–6. doi: 10.1128/AEM.64.6.2240-2246.1998 9603842 PMC106306

[62] Keiler K, Waller P, Sauer R. Role of a peptide tagging system in degradation of proteins synthesized from damaged messenger RNA. Science. 1996;271(5251):990–3. doi: 10.1126/science.271.5251.990 8584937

