# Supplementary figures and images for: Yersinia pseudotuberculosis doxycycline tolerance strategies include modulating expression of genes involved in cell permeability and tRNA modifications
Source: PLoS Pathog. 2022 May 16;18(5):e1010556. doi: 10.1371/journal.ppat.1010556 (PMC9135342; doi:10.1371/journal.ppat.1010556)

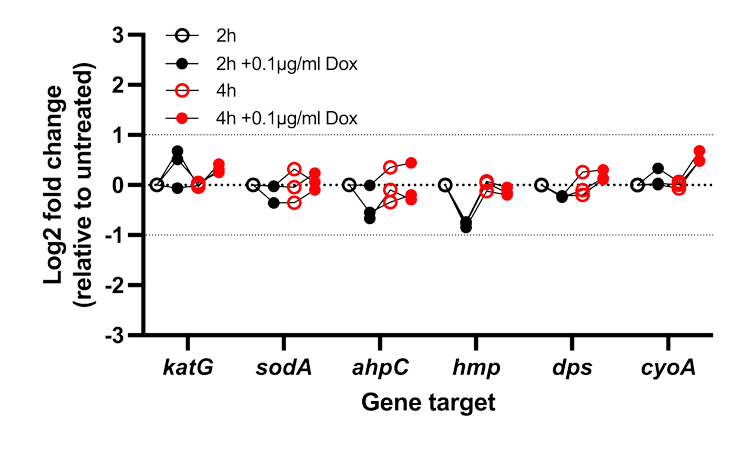

Supplement: S1 Fig — Cultures of WT Y. pseudotuberculosis were incubated in the presence or absence of 0.1μg/ml Dox, RNA was isolated and transcripts were detected by qRT-PCR. Log2 fold change values were calculated relative to untreated cells, horizontal dotted lines depict average values for untreated cells (0log2), and 2-fold changes. Data represents four biological replicates. (TIF) [file ppat.1010556.s001.tif]

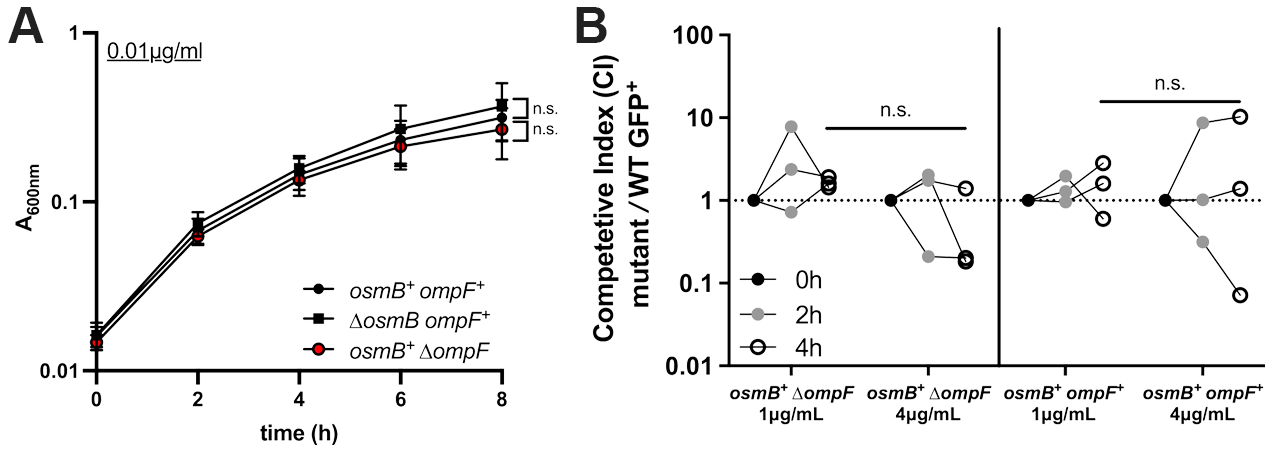

Supplement: S2 Fig — Exponential phase cultures of rescued strains (ΔosmB ompF+, osmB+ ΔompF, osmB+ ompF+) were treated with the indicated concentrations of Dox to assess (A) growth inhibition and (B) competitive survival. (A) Strains were incubated with 0.01μg/ml Dox and growth inhibition was assessed based on absorbance (A600nm) at the indicated timepoints (time: hours, h). Data represents the mean and standard deviation of four biological replicates. (B) Competitive survival in the presence of the indicated concentrations of Dox; rescued strains were tested alongside the WT GFP+ strain. Competitive index (CI): CFUs of the rescue/WT divided by the ratio of rescue/WT in the culture at time 0h. Values above 1 indicate the rescue preferentially survives, values less than 1 indicate the WT preferentially survives. Dotted line: value of 1, equal fitness. Dots: biological replicates, lines connect biological replicates sampled across the timepoints, three biological replicates shown. Statistics: Two-way ANOVA with Tukey’s multiple comparison test, (A): comparisons made relative to the osmB+ ompF+ strain; (B): comparisons made between 4h treatment CI values. n.s.: not significant. (TIF) [file ppat.1010556.s002.tif]

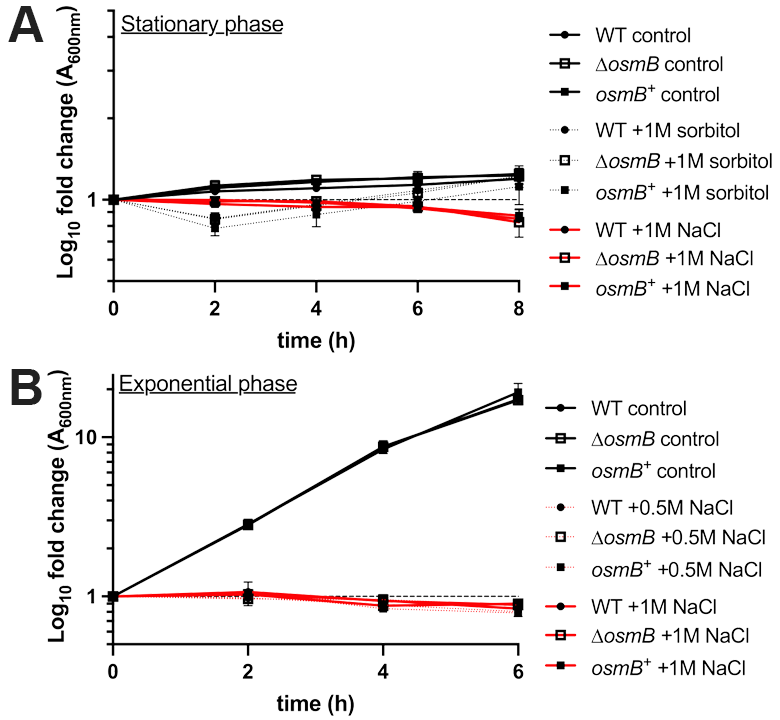

Supplement: S3 Fig — (A) Stationary and (B) exponential phase cultures of the WT, ΔosmB, and the osmB+ (osmB rescue) strains were incubated under the indicated high osmolarity conditions (1M sorbitol, 0.5M and 1M NaCl). Sensitivity was assessed based on absorbance (A600nm) at the indicated timepoints (time: hours, h). Data was normalized to the starting absorbance value of each culture (value of 1, dotted line), and is shown as log10 fold change. Controls depict vehicle controls. Data represents the mean and standard deviation of two biological replicates. Statistics: Two-way ANOVA (no statistical significance), post-tests not performed. (TIF) [file ppat.1010556.s003.tif]

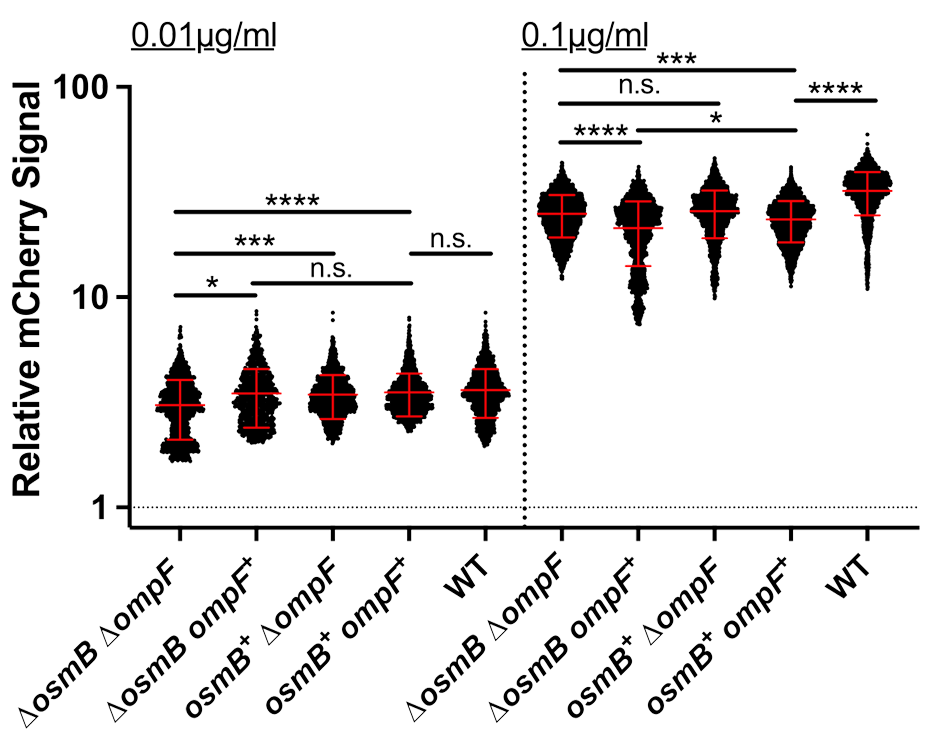

Supplement: S4 Fig — ΔosmB ΔompF, ΔosmB ompF+, osmB+ ΔompF, osmB+ ompF+, and WT strains transformed with the TetON reporter plasmid were incubated with the indicated concentrations of Dox for 4h. mCherry fluorescence was detected within individual cells by fluorescence microscopy. Relative mCherry was calculated by normalizing to a WT untreated average cell value (imaged in parallel). Data represents the mean and standard deviation of three biological replicates. Statistics: Kruskal-Wallis one-way ANOVA, Dunn’s post-test. ****p < .0001, ***p < .001, *p < .05, n.s.: not significant. (TIF) [file ppat.1010556.s004.tif]
